# NS1 of Duck Tembusu Virus Interacts with CCT2: Reduced CCT2 Recovery in LC3C Co-Immunoprecipitation In Vitro and the Proviral Role of CCT2 in DTMUV Infection

**DOI:** 10.3390/vetsci13090979

**Published:** 2026-09-17

**Authors:** Zekun Yang, Haixia Feng, Qiongjie Wang, Chengwei Xiang, Yin Han, Fei Xu, Mengyao Jing, Hongmei Li, Jianhua Liang, Xianming He, Ruiai Chen, Ting Xiong

**Affiliations:** 1 Zhaoqing Branch of Guangdong Laboratory of Lingnan Modern Agricultural Science and Technology, Zhaoqing 526238, China; 2 College of Veterinary Medicine, South China Agricultural University, Guangzhou 510642, China; 3 Key Laboratory of Biotechnology and Bioproducts Development for Animal Epidemic Prevention, Ministry of Agriculture and Rural Affairs, Zhaoqing 526238, China

**Keywords:** Duck Tembusu virus, NS1, CCT2, ubiquitin, autophagy

## Abstract

Regulation of Duck Tembusu virus (DTMUV) non-structural protein 1 (NS1) remains unclear. Exogenous and endogenous co-immunoprecipitation (Co-IP) assays demonstrated an association between the T-complex protein 1 subunit 2 (CCT2) and NS1. Further experiments mapped the NS1 region required for assembly of this complex to amino acid residues 172-352 of NS1.DTMUV increased CCT2 expression; CCT2 overexpression increased NS1 accumulation, whereas CCT2 knockdown delayed NS1 and envelope (E) protein accumulation. Although NS1 also associated with chaperonin-containing T-complex protein 1 subunit 6 (CCT6), CCT6 knockdown did not significantly alter NS1 messenger RNA (mRNA) or clearly change NS1 protein abundance. NS1 underwent lysine 48 (K48)- and lysine 63 (K63)-linked polyubiquitination. Increasing tripartite motif-containing protein 21 (TRIM21) reduced NS1 abundance and enhanced NS1-associated ubiquitin signals; the proteasome inhibitor MG132 promoted NS1 accumulation, which was highest with CCT2 co-expression, supporting proteasome-related turnover and CCT2-associated stabilization without establishing direct TRIM21 ubiquitination. CCT2 interacted with microtubule-associated protein 1 light chain 3C (LC3C), and DTMUV infection was associated with reduced late-stage lipidated LC3 (LC3-II) abundance and SQSTM1 mRNA expression. In dose-gradient Co-IP experiments, increasing NS1 expression was associated with a dose-associated reduction in CCT2 recovery in LC3C immunoprecipitates; the concomitant reduction in immunoprecipitated LC3C across the NS1 dose gradient precludes conclusions regarding direct competition or a binding capacity-dependent mechanism. These findings indicate that CCT2 is a DTMUV-associated host factor capable of stabilizing intracellular NS1 protein and promoting the production of infectious DTMUV progeny. CCT2 contributes to the homeostasis of NS1 protein.

## Simple Summary

Regulation of Duck Tembusu virus (DTMUV) non-structural protein 1 (NS1) remains poorly understood. Exogenous and endogenous co-immunoprecipitation assays identified that the chaperonin CCT2 exists in association with NS1, and the NS1 region required for assembly of their complex was mapped to residues 172–352. DTMUV infection elevates cellular CCT2 expression. CCT2 overexpression increases NS and envelope protein accumulation, whereas CCT2 knockdown delays viral protein production. Though CCT6 also binds NS1, silencing CCT6 hardly alters NS1 mRNA or protein levels. NS1 bears K48- and K63-linked polyubiquitin chains; CCT2 stabilizes NS1 against proteasomal breakdown. CCT2 forms an intracellular complex with LC3C. Under overexpression conditions, increasing NS1 expression was associated with reduced CCT2 recovery in LC3C immunoprecipitates. This work identifies CCT2 as a proviral host factor for DTMUV and highlights an association between NS1 expression and altered CCT2 recovery in LC3C immunoprecipitates.

## 1. Introduction

Duck Tembusu virus (DTMUV) is a member of the Flaviviridae family and exhibits genomic organization and replication mechanisms highly conserved among flaviviruses. The viral genome encodes three structural proteins—namely, the envelope (E), pre-membrane (prM), and capsid (C) proteins—as well as seven non-structural proteins (NS1, NS2A, NS2B, NS3, NS4A, NS4B, and NS5) [1]. Accumulating research demonstrates that the NS1 protein is essential for the assembly and functional development of the viral replication complex. For instance, soluble NS1 protein recruits and activates the C1s protease to cleave C4, while simultaneously binding to the complement regulatory protein C4BP, collectively inhibiting the activation of the classical and lectin pathways [2]. This inactivation of the complement system protects both the virus and infected cells, thereby facilitating viral replication and contributing to severe disease progression [3].

The CCT2 subunit of the TCP-1 ring complex (TRiC) chaperonin complex plays a critical role in suppressing protein misfolding and aggregation; however, its ability to stabilize substrate proteins is not yet fully elucidated [4]. Notably, CCT2 has recently been identified as a novel selective autophagy receptor for aggrephagy, capable of directly recognizing aggregation-prone proteins through its apical domain and interacting with ATG8 family proteins via a non-canonical VLIR motif. This interaction facilitates the degradation of pathogenic, insoluble protein aggregates that are refractory to clearance by conventional ubiquitin-dependent receptors such as p62 [5]. Consequently, CCT2 mediates the selective autophagic clearance of solid protein aggregates in a ubiquitin-independent manner.

Ubiquitination is a crucial post-translational modification that covalently attaches ubiquitin molecules to substrate proteins through an E1-E2-E3 enzyme cascade, thereby precisely regulating protein stability, activity, and localization [6]. Its most well-defined function is to form K48-linked polyubiquitin chains, mediating the degradation of target proteins by the 26S proteasome to maintain cellular homeostasis [7]. Additionally, other linkage types, such as K63-linked ubiquitination, are widely involved in non-degradative processes, such as signal transduction [8]. The role of the ubiquitination system in viral replication is complex. For instance, TRIM21 inhibits hepatitis B virus (HBV) replication during the early infection stage by directly triggering K48-linked ubiquitin chains and subsequent degradation of the viral HBx protein [9]. Conversely, in the case of Seneca Valley virus A, the host ubiquitin-conjugating enzyme UBE2L6 stabilizes the viral 3D polymerase through ubiquitination [10].

Autophagy is an evolutionarily conserved intracellular degradation and recycling system that delivers cytoplasmic components to lysosomes for degradation via double-membrane autophagosomes [11]. In antiviral immunity, autophagy has been recognized as an important cell-autonomous defense mechanism capable of selectively degrading viral components, a process known as “viral autophagy”. Research indicates that sorting nexin 5 (SNX5) specifically mediates virus-induced autophagy, thereby broadly suppressing the replication of various viruses including Zika virus (ZIKV), West Nile virus (WNV), and herpes simplex virus type 1 (HSV-1) [12].

In this study, we aimed to identify host factors associated with DTMUV NS1 and to characterize the relationship between NS1, CCT2, and viral replication. We further examined the association of CCT2 with NS1 protein stability and ubiquitin–proteasome-related regulation, and assessed whether NS1 expression was associated with changes in CCT2–LC3C co-immunoprecipitation. These experiments were designed to clarify the potential role of CCT2 in DTMUV infection and to provide a basis for future investigation of NS1–CCT2-mediated host–virus interactions.

## 2. Materials and Methods

### 2.1. Cell Lines, Virus and Viral Infections

Human embryonic kidney cells (HEK293T), Syrian hamster kidney cells (BHK-21), and chicken fibroblast cells (DF-1) were provided by the Zhaoqing Branch Center of Guangdong Laboratory for Lingnan Modern Agricultural Science and Technology, cultured in Dulbecco’s Modified Eagle Medium (DMEM;Gibco, Thermo Fisher Scientific, Waltham, MA, USA) or DMEM/F-12 (Procell Life Science & Technology Co., Ltd., Wuhan, China) supplemented with 10% fetal bovine serum (FBS; Gibco, Thermo Fisher Scientific, Waltham, MA, USA), 2 mM L-glutamine (Gibco, Thermo Fisher Scientific, Waltham, MA, USA), and 1% penicillin-streptomycin (Sigma-Aldrich Co. LLC, St. Louis, MO, USA). All cells were maintained at 37 °C under 5% CO_2_. The DTMUV QY17 strain (GenBank Accession No. MT447092) was preserved by the Zhaoqing Branch Center of Guangdong Laboratory for Lingnan Modern Agriculture Science and Technology [13]. In the virus infection experiment, DF-1 cells were inoculated into 6-well plates and then infected with DTMUV at a multiplicity of infection (MOI) of 1 unless otherwise indicated. After 1.5 h of virus adsorption at 37 °C, the inoculation medium was discarded, and the cells were washed three times with phosphate-buffered saline (PBS; Gibco, Thermo Fisher Scientific, Waltham, MA, USA). Fresh DMEM/F-12 medium containing 1% bovine serum albumin (BSA; Sigma-Aldrich Co. LLC, St. Louis, MO, USA) was added, and the plates were placed in a 37 °C, 5% CO_2_ incubator for further culture.

### 2.2. Construction of Recombined Plasmids

Using the specific primers listed in Appendix A Table A1, the CCT2 gene fragment was amplified by PCR with the genomic DNA of HEK293T cells as the template. Then, it was seamlessly cloned into the pCMV-Flag vector backbone using the pEASY^®^-Basic cloning kit (TransGen Biotech Co., Ltd., Beijing, China) to construct the expression vectors of pCMV-Flag-CCT2 and other TCP-1 molecular chaperone family members. RNA was extracted from DTMUV-infected DF-1 cells, and the full-length NS1 open reading frame was obtained by RT-PCR amplification. Subsequently, it was inserted into the pCMV-Myc vector through homologous recombination to construct pCMV-Myc-NS1 and its truncated mutants.

### 2.3. Analysis of Potential Proteins Interacting with DTMUV NS1 in Host Cells by IP-MS

HEK293T cells were transfected with either the empty eukaryotic expression vector pCMV-Myc or the pCMV-Myc-NS1 plasmid. After incubation for 24 h at 37 °C, cells were lysed in 1 mL of NP-40 lysis buffer. The cell lysates were immunoprecipitated overnight at 4 °C using anti-Myc antibody-conjugated agarose beads. On the following day, the beads were washed three times and subjected to co-immunoprecipitation (Co-IP) coupled with liquid chromatography–tandem mass spectrometry (LC-MS/MS) analysis.

### 2.4. Co-Immunoprecipitation and Western Blotting

The interaction between CCT2 and NS1 proteins was validated by Co-IP and Western blot analysis. HEK293T cells seeded in 6-well plates were transfected with the indicated plasmids and incubated for 24 h. Cells were lysed in NP-40 lysis buffer on ice for 30 min, followed by centrifugation at 12,000× *g* for 8 min at 4 °C. A 40 μL aliquot of the supernatant was retained as input control, while the remaining lysate was incubated overnight at 4 °C with EZview™ Red Anti-c-Myc Affinity Gel (Sigma-Aldrich (Merck KGaA), St. Louis, MO, USA). The beads were washed five times with NP-40 lysis buffer, and bound proteins were eluted by boiling in 2× SDS sample buffer. Proteins were separated by SDS-PAGE and transferred onto nitrocellulose membranes (Merck Millipore, Burlington, MA, USA). Membranes were blocked with 5% skim milk in TBST for 1 h at room temperature, then probed with primary antibodies against Flag (Nulen Biotech, Shanghai, China), Myc (Nulen Biotech, Shanghai, China), CCT2 (Proteintech Group, Wuhan, China), CCT6 (Proteintech Group, Wuhan, China), α-Tubulin (Proteintech Group, Wuhan, China), GAPDH (Proteintech Group, Wuhan, China), β-Actin (Proteintech Group, Wuhan, China), as well as NS1 and E proteins, which were prepared and stored in our laboratory. After three washes with TBST, membranes were incubated with horseradish peroxidase (HRP)-conjugated goat anti-mouse or anti-rabbit IgG secondary antibodies (1:5000 dilution) for 1 h at room temperature. Following additional washes (three times), protein signals were detected using an ECL chemiluminescent substrate (Beyotime Biotechnology, Shanghai, China) and visualized on an ImageQuant 800 imaging system (Cytiva, Marlborough, MA, USA). Mouse monoclonal antibodies against DTMUV NS1 and E were generated by immunizing BALB/c mice with recombinant NS1 or E protein, followed by fusion of splenocytes with SP2/0 myeloma cells and screening of antigen-specific hybridomas maintained at the Zhaoqing Branch Center. Both antibodies were used for Western blotting at 1:5000. For tagged-protein Co-IP assays, cells transfected with the corresponding empty tag vector were processed in parallel using equal lysate and bead amounts and identical incubation, washing, elution, and immunoblotting procedures to assess nonspecific binding to the tag and magnetic matrix.

For the NS1 dose-gradient Co-IP assay, the amounts of Flag-CCT2 and Myc-LC3C plasmids were kept constant. The indicated CCT2-binding-deficient NS1 truncation mutant was included as a negative interference control, whereas full-length Flag-NS1 plasmid was transfected at 0.5, 1, 2, or 3 μg before anti-Myc immunoprecipitation. For the dose-gradient Co-IP assay, an empty vector plasmid was used to equalize total transfected DNA amount across all groups to eliminate variations in transfection efficiency and cellular toxicity caused by unequal plasmid input.

### 2.5. Endogenous Interaction Between DTMUV NS1 and Host CCT2 Protein

To investigate the endogenous interaction between DTMUV NS1 and the host CCT2 protein under physiological conditions, BHK-21 cells were infected with DTMUV at a multiplicity of infection (MOI) of 1, with the viral E protein serving as a bait control. Cell lysates were incubated with 2.5 μL of monoclonal antibodies specific to NS1 or E protein for 4 h at 4 °C, followed by overnight incubation at 4 °C with pre-equilibrated Protein A/G agarose beads. Co-immunoprecipitation assays were subsequently performed, using monoclonal antibodies against NS1, CCT2, or E protein as primary antibodies, and horseradish peroxidase (HRP)-conjugated goat anti-mouse or anti-rabbit IgG as the secondary antibody. The E-protein control was processed under the same infection, lysis, immunoprecipitation, washing, and detection conditions as the NS1 sample.

### 2.6. Cycloheximide Chase Assay for NS1 Protein Stability

To determine the stability of the NS1 protein, a cycloheximide (CHX) chase assay was conducted. HEK293T cells were seeded in 6-well plates and co-transfected with plasmids encoding NS1, CCT2, Ub, and TRIM21 using a transfection reagent. Twenty-four hours post-transfection, the medium was replaced with complete medium containing 100 μg/mL CHX (MedChemExpress) to inhibit protein synthesis, with control wells receiving an equal volume of DMSO vehicle. Cells were harvested at 0, 2, 4, and 6 h after CHX treatment. The harvested cells were lysed using ice-cold RIPA buffer supplemented with protease and phosphatase inhibitors. Following centrifugation, the supernatants were collected, and protein concentrations were determined using the BCA method. The expression levels of NS1 and a loading control protein (GAPDH) were analyzed by Western blot. Band intensities were quantified using ImageJ 1.50b (National Institutes of Health, Bethesda, MD, USA). The relative remaining levels of NS1 at each time point were calculated, with the 0 h sample set as the baseline (100%). The final DMSO concentration was maintained consistently among all corresponding experimental groups to exclude solvent-related effects.

For the TRIM21 dose–response experiment, a fixed amount of Myc-NS1 plasmid was co-transfected with 0, 500, 800, or 1000 ng of Flag-TRIM21 plasmid, and cells were harvested 24 h later. For proteasome-inhibition experiments, NS1-expressing cells received CCT2 or empty vector and were treated with MG132 or the corresponding DMSO vehicle before Western blotting or anti-Myc immunoprecipitation of NS1-associated HA-ubiquitin.

### 2.7. RNA Extraction and Quantitative Real-Time RT-PCR

Total RNA was extracted from HEK293T and BHK-21 cells using TRIzol™ Reagent (Invitrogen, Thermo Fisher Scientific, Waltham, MA, USA) according to the manufacturer’s instructions, followed by cDNA synthesis with HiScript^®^ III RT SuperMix (Vazyme Biotech Co., Ltd., Nanjing, China). RT-qPCR was performed on a QuantStudio™ 3 Real-Time PCR System(Applied Biosystems (Thermo Fisher Scientific), Foster City, CA, USA) using ChamQ Universal SYBR qPCR Master Mix (Vazyme Biotech Co., Ltd., Nanjing, China) under standard thermal cycling conditions: 95 °C for 30 s (initial denaturation), followed by 40 cycles of 95 °C for 10 s and 60 °C for 30 s. A melting-curve analysis was performed after amplification to verify product specificity. Gene expression levels were normalized to GAPDH and calculated using the 2^−ΔΔCt^ method relative to untreated controls, with primer sequences listed in Appendix A Table A2 [14].

### 2.8. RNA Interference Assay

BHK-21 cells were seeded in 12-well plates at 60–70% confluence and transfected with gene-specific siRNAs (siCCT2-1: 5′-GCUCACAGUGAAGGCCAUAUATT-3′; siCCT2-2: 5′-GCUACCAUUCUCAAGAACAUUTT-3′; siCCT2-3: 5′-GCUGUAGCAAUGGAGUCGUUUTT-3′) or negative control siRNA (siNC) using Lipofectamine RNAiMAX (Invitrogen, USA) according to the manufacturer’s instructions. Samples were harvested 24 h post-transfection, and knockdown efficiency was assessed by RT-qPCR analysis of CCT2 mRNA levels and Western blotting of CCT2 protein expression using rabbit anti-CCT2 polyclonal antibodies. All siRNAs were designed and synthesized by Sangon Biotech (Shanghai, China). Among the three CCT2 siRNAs, siCCT2-3 produced the strongest reduction at both the mRNA and protein levels and was selected for subsequent functional experiments. Three independent siRNAs targeting CCT6 (siCCT6-1: 5′-CAUUGAAGAUAGAGUUAAATT-3′; siCCT6-2: 5′-GGUCUUGUCUAUGAGUAUATT-3′; siCCT6-3: 5′-GAGACAUCAAGCUUACUAATT-3′) were evaluated in parallel, and the siRNA producing the strongest knockdown was selected for the infection time-course experiment.

### 2.9. Statistical Analysis

Molecular cloning was performed using SnapGene^®^ 3.2.1 software. Statistical analyses and graphical visualization were conducted using GraphPad Prism 8.0, and image processing was carried out with Adobe Photoshop 2022. All experiments were performed with at least three independent biological replicates (*n* ≥ 3), and technical replicates within each experiment were averaged before statistical analysis. Data are presented as mean ± standard deviation (SD). Student’s *t*-test was used for two-group comparisons. One-way ANOVA was applied for single-factor multiple-group comparisons, whereas two-way ANOVA was used for time-course experiments with two experimental variables. A *p*-value ≤ 0.05 was considered statistically significant. For whole-cell-lysate Western blot quantification, band intensity of target proteins was normalized to the corresponding loading-control band (GAPDH, tubulin or β-actin) within the same lane. For standard co-immunoprecipitation assays, the band intensity of co-precipitated prey protein was normalized to the band intensity of bait protein from the same IP lane. For the NS1 dose-gradient Co-IP assay, immunoprecipitated CCT2 signal was normalized against immunoprecipitated LC3C signal within each individual IP lane to correct for variation in LC3C pull-down efficiency. Relative values were calculated by setting the NS1 (1-177) group (NS1 truncation mutant deficient in CCT2-binding) to 1.0. Statistical tests including Student’s *t*-test and one-way ANOVA with appropriate post hoc multiple-comparison tests are indicated in corresponding figure legends.Significance symbols in figures are defined as: ns, not significant (*p* > 0.05); * *p* < 0.05; ** *p* < 0.01; *** *p* < 0.001; **** *p* < 0.0001.

### 2.10. Bioinformatic Analysis

Candidate NS1-interacting proteins obtained from LC-MS/MS were annotated against the UniProt database. Gene Ontology (GO, cellular component, molecular function, biological process) and KEGG pathway enrichment analyses were performed. Multiple-testing correction was applied using the false discovery rate (FDR). Terms with adjusted-*p* value (FDR) < 0.05 were regarded as significantly enriched. The STRING database was used for protein-protein interaction analysis, and the PPI network was visualized with Cytoscape v3.8.2 software.

### 2.11. Determination of Infectious Viral Titer by TCID_50_ Assay

BHK-21 cells were seeded in 96-well plates and cultured to 80–90% confluence. Cell-culture supernatants collected at indicated time points post-infection were serially 10-fold diluted with DMEM/F-12 medium supplemented with 2% FBS. Each dilution was added to 96-well plates with eight replicate wells per dilution. Cells were incubated at 37 °C under 5% CO_2_, and cytopathic effect (CPE) was monitored microscopically daily. TCID_50_ values were calculated using the Reed-Muench method. Viral titers were expressed as log_10_ TCID_50_/mL. All titration experiments were performed with three independent biological replicates.

## 3. Results

### 3.1. Bioinformatics Analysis of the Interaction Between DTMUV NS1 Protein and Host Cell Proteins

To identify host proteins that may interact with NS1, 293T cells were transfected with a Myc-tagged NS1 expression vector and an empty control vector, respectively. At 24 h post-transfection, IP was performed using an anti-Myc antibody, and the precipitated proteins were subjected to mass spectrometry analysis to identify potential interacting partners. Mass spectrometric profiling identified 537 putative NS1-interacting host proteins, with 42 exhibiting high-affinity binding characteristics. Proteins detected in the NS1 immunoprecipitation group but absent from or markedly enriched relative to the empty-vector control, with at least two unique peptides and a confidence score > 30, were retained as candidate interacting molecules for subsequent bioinformatics enrichment and network analysis. These candidate proteins were functionally annotated using the UniProt database and subjected to Gene Ontology (GO) enrichment analysis, COG functional annotation, KEGG enrichment analysis and construction of protein–protein interaction networks.

**Figure 1:**
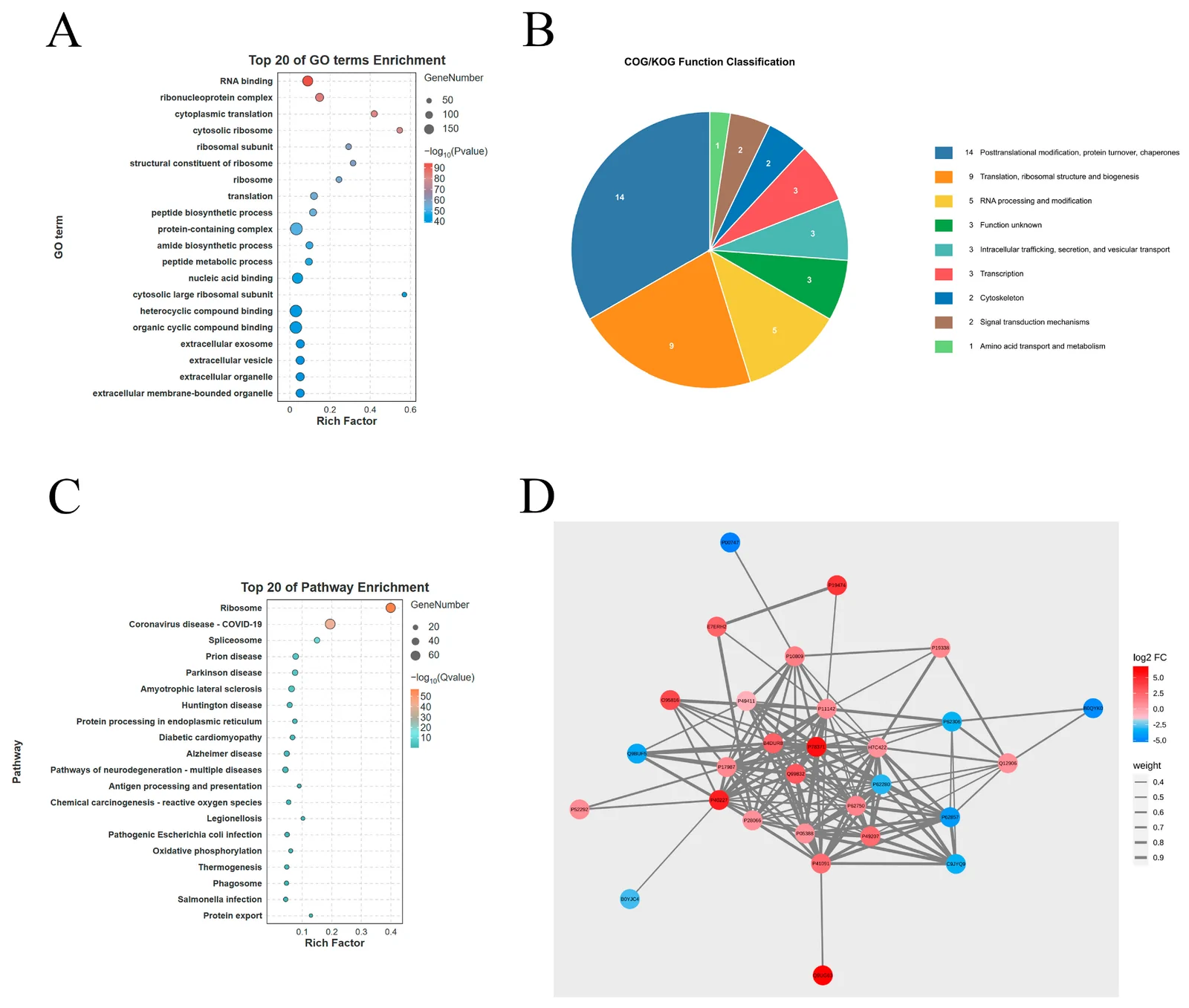
Bioinformatic analysis of interactions between DTMUV NS1 and host proteins in HEK293T cells. (**A**) GO annotation and enrichment analysis of NS1-interacting proteins. (**B**) COG functional classification of NS1-interacting host proteins. (**C**) KEGG pathway enrichment analysis for NS1-interacting proteins. (**D**) PPI network of cellular proteins interacting with NS1.

GO analysis classified the NS1-interacting proteins into three standard ontological domains: cellular component (CC), molecular function (MF), and biological process (BP). Notably, chaperone-related complexes accounted for 7.14% of the identified interactors, pointing toward a potential functional representation of protein-folding machinery among NS1-binding host factors (Figure 1A). COG functional annotation revealed three major categories: post-translational modification, protein folding, and chaperones (33.3%); translation and ribosome biogenesis (21.43%); and RNA processing and modification (11.9%). This functional profile reflects the broad functional repertoire of NS1-associated host proteins, covering protein homeostasis, translation, and RNA-metabolism-related factors (Figure 1B). KEGG enrichment analysis ranked the proteasome pathway as the fifth most significantly enriched category among NS1-interacting candidates (Figure 1C). This enrichment represents a statistical feature of the identified interactome, and further experimental work is required to define the functional relevance of the proteasome pathway during NS1-host interplay. Protein–protein interaction networks, constructed using the STRING database and visualized with Cytoscape, identified CCT2 as a high-confidence NS1 interactor, warranting further investigation into its interaction mechanism and functional impact during DTMUV infection (Figure 1D).

### 3.2. Verification of the Interaction of DTMUV NS1 Protein with Exogenous and Endogenous CCT2 and Mapping the Interaction Domain

**Figure 2:**
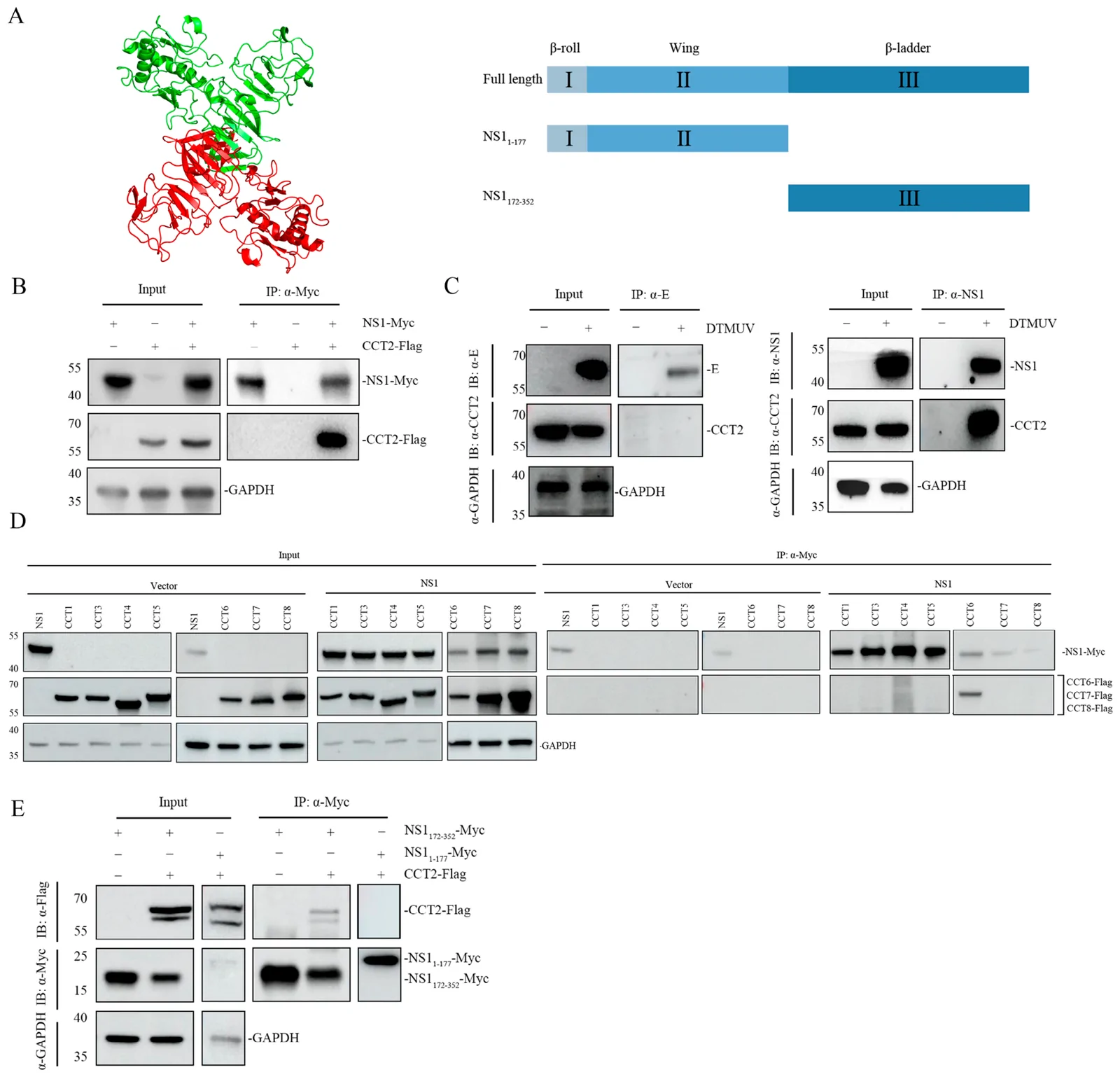
Co-immunoprecipitation assays for DTMUV NS1 and CCT family proteins. (**A**) Schematic representation of full-length NS1 and truncated NS1 constructs, showing domain organization (β-roll, Wing, β-ladder),NS1 forms a homodimer, with red and green representing its two monomers. (**B**) HEK293T cells were co-transfected with Myc-NS1 and Flag-CCT2. Cell lysates were subjected to anti-Myc co-immunoprecipitation and immunoblot analysis. (**C**) BHK-21 cells were infected with DTMUV (MOI = 1). Cell lysates were immunoprecipitated using anti-NS1 or anti-E monoclonal antibody, followed by immunoblotting. Anti-E served as bait control. (**D**) HEK293T cells co-expressing Myc-NS1 and individual Flag-tagged TCP-1/CCT subunits (CCT1–CCT8). Anti-Myc agarose beads were used for immunoprecipitation. (**E**) HEK293T cells co-transfected with Flag-CCT2 and Myc-tagged NS1 truncation mutants (NS1_1–177_, NS1_172–352_). Anti-Myc co-IP was performed to assess protein binding. GAPDH served as loading control for input samples across panels (**B**–**E**).

To verify the interaction between DTMUV NS1 and CCT family subunits, we constructed Flag-tagged expression plasmids for all CCT subunits. HEK293T cells were co-transfected with pCMV-NS1-Myc and each Flag-tagged CCT subunit construct. Cell lysates were subjected to anti-Myc agarose-based co-immunoprecipitation followed by Western blot analysis. Negative controls were included, and the results confirmed that Myc-tagged NS1 specifically interacts with Flag-tagged CCT2 and CCT6 (Figure 2B,D), whereas no binding was detected between NS1 and other CCT subunits (Figure 2D). To further confirm this interaction under virus-infection conditions, BHK-21 cells were infected with DTMUV for endogenous co-IP assays. The DTMUV envelope (E) protein served as a bait-matched negative control. Endogenous CCT2 was co-precipitated with NS1 in DTMUV-infected cells, while no signal was recovered in the E-protein immunoprecipitation group (Figure 2C). These findings demonstrate that CCT2 is detected in intracellular complexes with NS1 under both overexpression and virus-infection settings. A standard IgG isotype control was omitted because the 55 kDa IgG heavy-chain band would interfere with CCT2 detection; in addition, stocks of NS1-specific monoclonal antibody were exhausted after assay optimization and replicate experiments. Therefore, DTMUV E protein was used as an alternative negative control under identical experimental conditions. Recovery of CCT2 in NS1 immunocomplexes, but not in E-protein precipitates, supports the specificity of this interaction. To map the CCT2-binding region within NS1, two NS1 truncation constructs were generated: pCMV-myc-NS1(1–177) encoding the β-roll and Wing domains, and pCMV-myc-NS1(172–352) corresponding to the β-ladder domain (Figure 2A). These constructs were co-transfected with pXJ40-CCT2-Flag into HEK293T cells, and co-IP was performed at 24 h post-transfection. CCT2-Flag was pulled down only by NS1(172–352)-Myc but not by NS1(1–177)-Myc (Figure 2E). Two CCT2-immunoreactive bands were detected under some conditions. Previous work has identified CCT2 as a physiological substrate of RSK/p90 and S6K/p70, with Ser260 reported as a major RSK phosphorylation site, suggesting that phosphorylation-dependent mobility changes may contribute to the banding pattern [15]. However, the molecular identities of the two bands were not directly determined in the present study. Taken together, these results map the CCT2-binding interface to the β-ladder domain of NS1, spanning amino acid residues 172–352.

### 3.3. CCT2 Overexpression Increases Intracellular Viral Protein Accumulation and Enhances Infectious DTMUV Progeny Production

**Figure 3:**
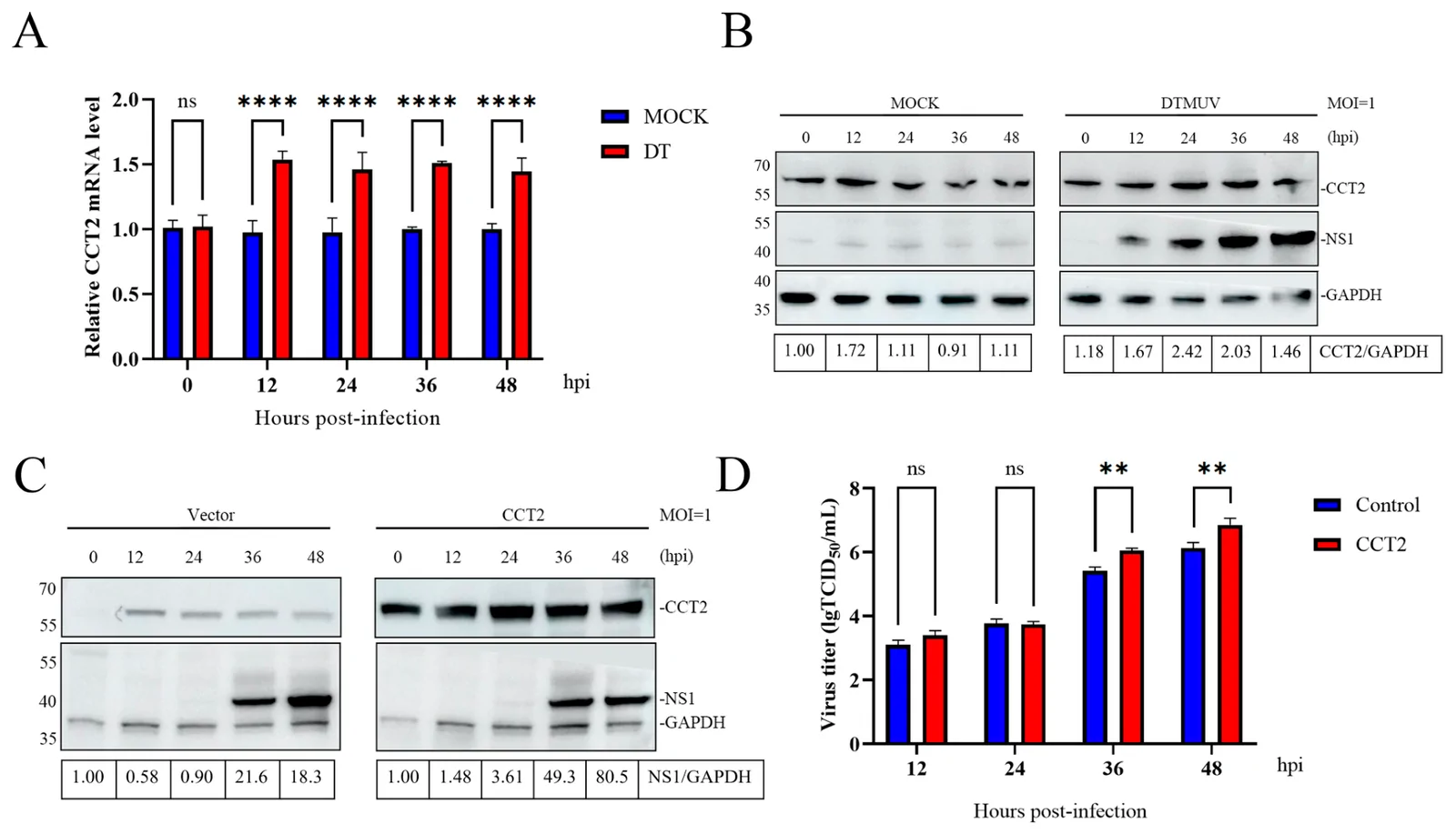
RT-qPCR, immunoblot, and TCID_50_ assays for DTMUV-infected BHK-21 cells with or without CCT2 overexpression. (**A**) BHK-21 cells were infected with DTMUV (MOI = 1) or mock-treated and harvested at designated time points for RT-qPCR measurement of CCT2 mRNA. (**B**) Whole-cell lysates from mock-treated and DTMUV-infected BHK-21 cells were subjected to immunoblot detection of CCT2 and NS1. (**C**) BHK-21 cells transfected with pXJ40-Flag-CCT2 or empty control vector were infected with DTMUV 24 h post-transfection. Cells were collected at the indicated time points for immunoblot analysis. (**D**) Cell-culture supernatants from vector-control and CCT2-overexpressing infected cells were collected for TCID_50_ measurement; viral titers are reported as log_10_ TCID_50_/mL. GAPDH served as loading control for immunoblot samples across panels (**B**,**C**). All data represent the mean ± SD from three independent biological replicates. Statistical significance was determined using two-way ANOVA; ** *p* < 0.01; **** *p* < 0.0001; ns, not significant (*p* > 0.05).

To investigate the expression kinetics of CCT2 during DTMUV infection and the impact of CCT2 overexpression on DTMUV replication, BHK-21 cells were either infected with DTMUV at an MOI of 1 or mock-treated, and harvested at 0, 12, 24, 36, and 48 hpi, respectively, and the expression of CCT2 was assessed at both mRNA and protein levels by RT-qPCR and Western blot. Compared with mock-treated control cells, CCT2 mRNA expression was markedly upregulated from 12 hpi and sustained throughout the detection period, indicating that DTMUV infection induces transcriptional upregulation of CCT2 (Figure 3A). At the protein level, CCT2 expression was elevated at 24–36 hpi, peaking at 24 hpi and returning to above the baseline by 48 hpi (Figure 3B). This pattern of initial increase followed by decline was consistent with the mRNA expression dynamics. The effect of CCT2 overexpression on DTMUV replication was then investigated by transfection of BHK-21 cells with plasmid pXJ40-flag-CCT2. At 24 h post-transfection, cells were infected with DTMUV and harvested at 0, 12, 24, 36 and 48 hpi, respectively, for Western blot analysis. CCT2 overexpression increased NS1 protein accumulation, with the most pronounced effects observed at 36 and 48 hpi (Figure 3C). To further evaluate whether CCT2 modulates the production of infectious virions, cell-culture supernatants collected at corresponding time points were subjected to TCID_50_ measurement (Figure 3D). No significant differences in infectious viral yields were observed at 12 and 24 hpi. At 36 and 48 hpi, overexpression of CCT2 significantly elevated infectious DTMUV titers. These data demonstrate that CCT2 overexpression augments both intracellular viral-protein abundance and extracellular infectious virus production at the late infection stage.

### 3.4. Comparative Analysis of CCT2 and CCT6 Knockdown During DTMUV Infection

**Figure 4:**
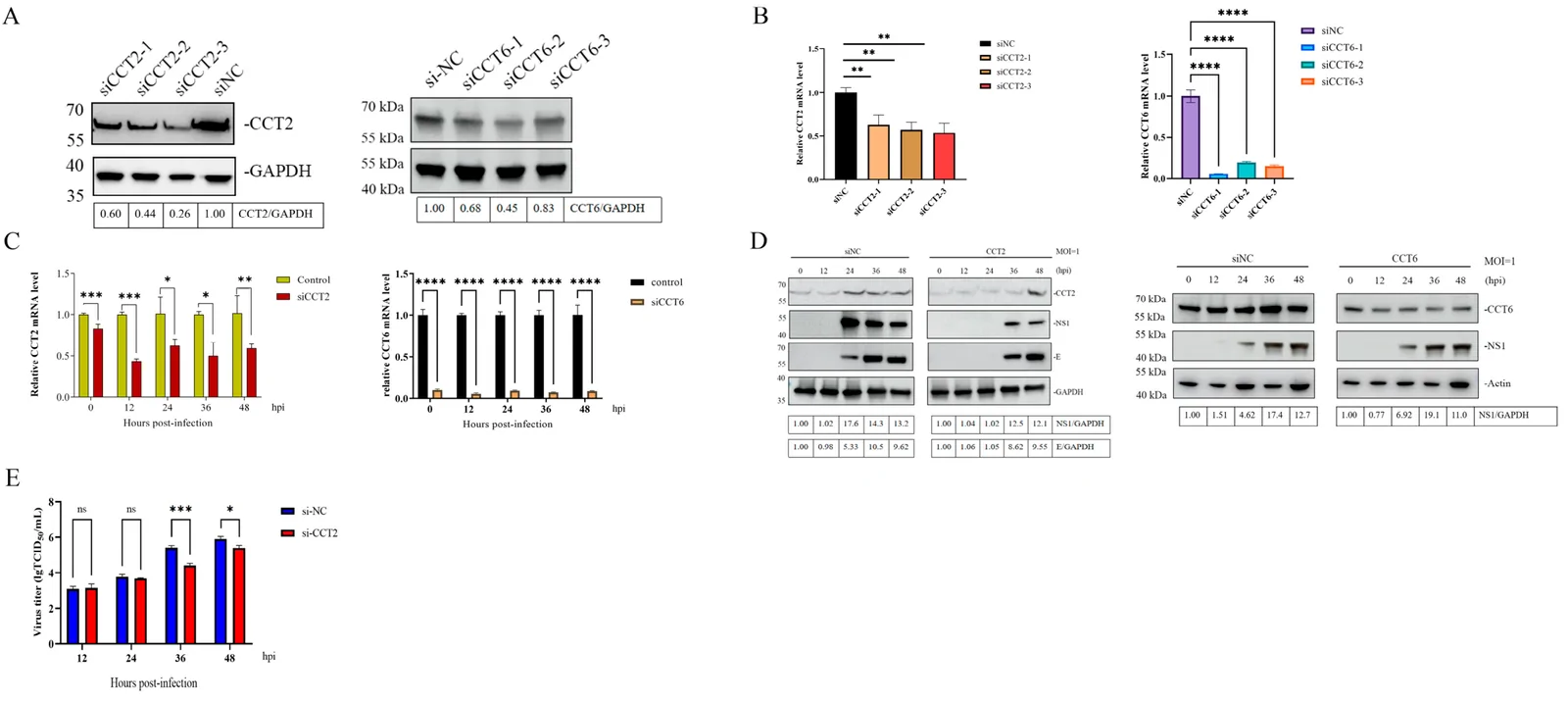
RNA-interference-based assays in DTMUV-infected BHK-21 cells. (**A**) Immunoblot analysis of CCT2 and CCT6 protein levels following transfection of three distinct siRNA constructs targeting each gene. (**B**) RT-qPCR quantification of CCT2 and CCT6 mRNA abundance after transfection of the indicated siRNAs. (**C**) Time-course RT-qPCR to assess the persistence of CCT2 or CCT6 knockdown in DTMUV-infected cells (MOI = 1). (**D**) Immunoblot detection of viral proteins in si-treated, DTMUV-infected BHK-21 cells. NS1 and E were assessed for CCT2 knockdown; only NS1 was analyzed for CCT6 knockdown. (**E**) TCID_50_ measurement of infectious virus in culture supernatants harvested from si-NC- and si-CCT2-3-transfected DTMUV-infected cells; viral titers are reported as log_10_ TCID_50_/mL. Loading-control proteins were used for normalization of immunoblot band intensities across panels (**A**,**B**,**D**). All data represent the mean ± SD of three independent biological replicates. Statistical analysis was performed using two-way ANOVA; * *p* < 0.05; ** *p* < 0.01; *** *p* < 0.001; **** *p* < 0.0001; ns, not significant (*p* > 0.05).

To further investigate the impact of CCT2 on DTMUV replication, three siRNA constructs targeting CCT2 (siCCT2-1, siCCT2-2, and siCCT2-3) were designed and transfected into BHK-21 cells, with siNC included as a negative control. Each group was performed in triplicate. At 24 h post-transfection, Western blot and qRT-PCR analyses confirmed that all three siRNAs significantly reduced the endogenous CCT2 expression at both mRNA and protein levels, compared to the siNC control group (Figure 4A,B). Notably, transfection of cells with siCCT2-3 achieved an approximately 50% knockdown efficiency at the mRNA level and over 90% reduction at the protein level, and was therefore selected for subsequent experiments (Figure 4A,B). BHK-21 cells transfected with siCCT2-3 were then infected with DTMUV at 24 h post-transfection, and harvested at 0, 12, 24, 36, and 48 hpi, respectively. RT-qPCR analysis demonstrated a significant reduction in CCT2 at the mRNA level in the knockdown group (Figure 4C). Western blot analysis revealed delayed viral replication kinetics in CCT2-knockdown cells (Figure 4D). While NS1 protein expression was detectable in control cells at 24 hpi, its expression in the knockdown group was delayed until 36 hpi (Figure 4D). Western blot analysis of DTMUV structural protein E also showed a similar expression pattern as NS1 (Figure 4D). We further measured infectious virus yields of culture supernatants using the TCID_50_ assay (Figure 4E). Consistent with the intracellular viral-protein kinetics, silencing endogenous CCT2 significantly reduced infectious DTMUV progeny titers at 36 and 48 hpi, whereas no obvious difference was observed at early time points (12, 24 hpi). Collectively, these data show that loss-of-function of CCT2 delays intracellular viral-protein appearance and suppresses the release of extracellular infectious virions.

CCT6 was evaluated in parallel using three independent siRNAs. The selected CCT6 siRNA maintained strong suppression from 0 to 48 hpi. CCT6 knockdown did not significantly alter NS1 mRNA at 12, 24, 36, or 48 hpi and did not produce a clear change in NS1 protein abundance. Thus, CCT6 knockdown did not measurably alter the examined molecular readouts under these conditions; however, infectious virus yield was not quantified for CCT6-knockdown cells, and a context-dependent or compensatory role of CCT6 cannot be excluded. The corresponding CCT6 siRNA screening, knockdown time course, and NS1 expression analyses are shown in Figure 4A–D.

### 3.5. Ubiquitin–Proteasome-Related Regulation of NS1 Abundance

**Figure 5:**
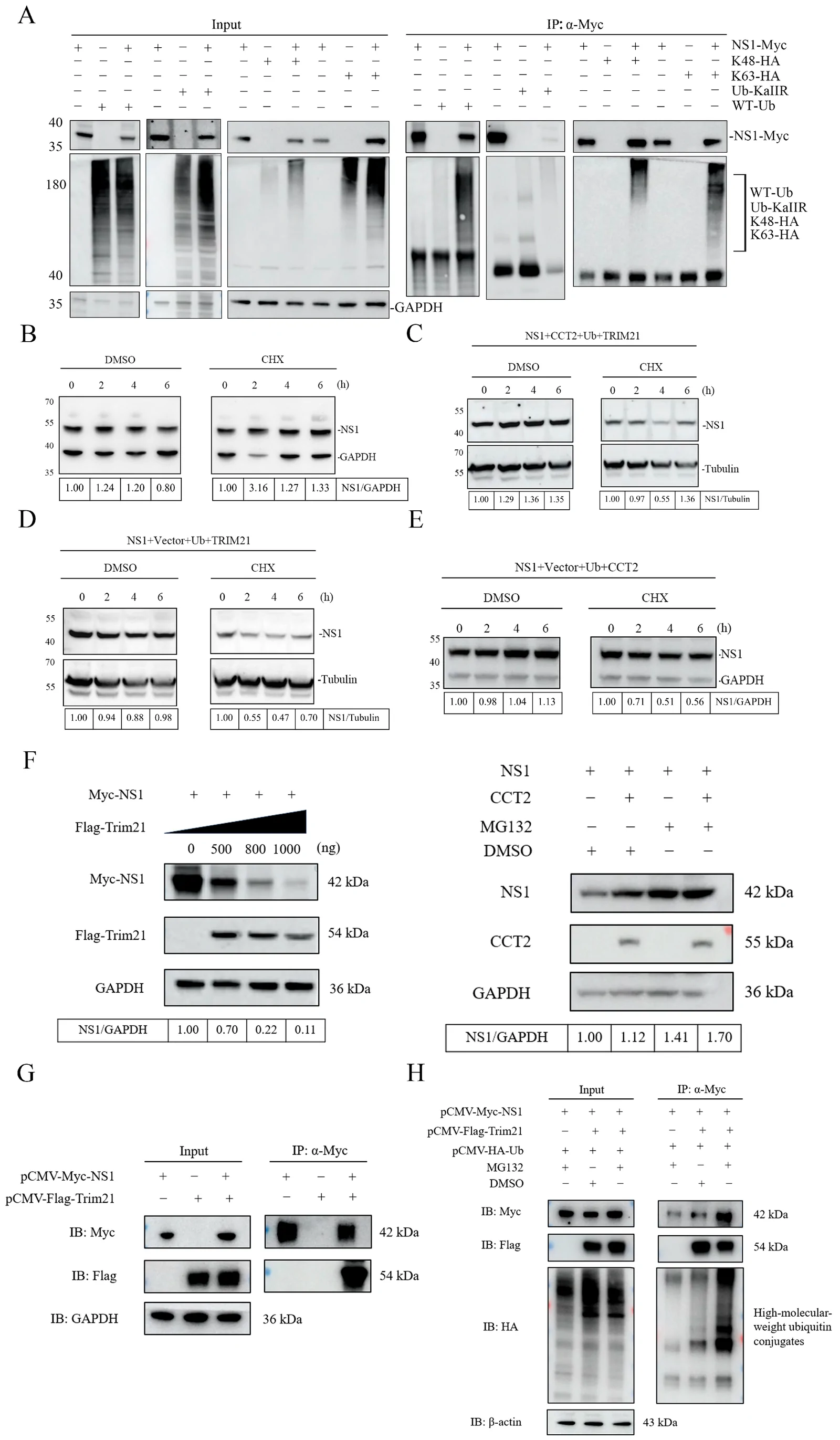
Ubiquitination and proteasome-related regulation of NS1 abundance. (**A**) Analysis of NS1-associated ubiquitination in HEK293T cells co-expressing Myc-NS1 with wild-type HA-Ub, K48-only HA-Ub, K63-only HA-Ub, or lysine-deficient HA-Ub (KallR). (**B**) CHX-chase analysis of NS1 abundance in DTMUV-infected BHK-21 cells. Cells infected with DTMUV (MOI = 1) were treated with CHX (100 μg/mL) or DMSO at 24 hpi and harvested at 0, 2, 4, and 6 h after treatment. NS1 signals were normalized to GAPDH. (**C**–**E**) CHX-chase analysis of NS1 abundance in transfected BHK-21 cells expressing: (**C**) Myc-NS1, Flag-CCT2, HA-Ub, and Flag-TRIM21; (**D**) Myc-NS1, HA-Ub, Flag-TRIM21, and empty vector; or (**E**) Myc-NS1, HA-Ub, Flag-CCT2, and empty vector. Cells were treated with CHX (100 μg/mL) at 24 h post-transfection and harvested at the indicated times. (**F**) Effects of increasing Flag-TRIM21 expression on Myc-NS1 abundance and effects of CCT2 expression and MG132 treatment on NS1 abundance. NS1 signals were normalized to GAPDH. (**G**) Co-immunoprecipitation analysis of Myc-NS1 and Flag-TRIM21 in HEK293T cells. (**H**) Analysis of NS1-associated HA-ubiquitin in the absence or presence of Flag-TRIM21 and MG132. Data represent the mean ± SD from three independent biological replicates.

To investigate whether CCT2 maintains NS1 protein stability through the ubiquitin–proteasome pathway, we first examined the ubiquitination modification of NS1. We constructed a wild-type HA-tagged ubiquitin plasmid (pXJ40-HA-ub) and three ubiquitin mutants: pXJ40-HA-ub(K48), pXJ40-HA-ub(K63), and pXJ40-HA-ub(-K), which only retains lysine at position 48, only retains lysine at position 63, or has all lysine residues mutated to arginine, respectively. Co-expression of NS1 with wild-type, K48-only, or K63-only ubiquitin showed that NS1 could be ubiquitinated, while ubiquitination of NS1 was completely abolished when all lysine residues of ubiquitin were mutated (Figure 5A). This experiment only demonstrates the ubiquitination profile of NS1 and does not involve CCT2. A cycloheximide (CHX) chase assay showed that NS1 undergoes progressive degradation over time in cells (Figure 5B). To further determine whether CCT2 affects NS1 stability, HEK293T cells were co-transfected with three different plasmid combinations: NS1 + CCT2 + Ub + TRIM21, NS1 + Ub + TRIM21, and NS1 + CCT2 + Ub. TRIM21 was selected as a well-characterized E3 ubiquitin ligase that mediates ubiquitination and proteasomal degradation of cellular and viral proteins [9]. After 24 h, cells were treated with CHX, and samples were collected at 0, 2, 4, and 6 h. Western blot was used to analyze the degradation rate of NS1. Reduced NS1 abundance was observed in the presence of ubiquitin and TRIM21 (Figure 5D), consistent with a TRIM21-associated effect on NS1 stability. CCT2 expression was associated with altered NS1 degradation kinetics (Figure 5C), suggesting a possible stabilizing effect. In the absence of TRIM21 (NS1 + CCT2 + Ub), the degradation rate of NS1 was moderate (Figure 5E), supporting that CCT2 contributes to NS1 stabilization. Additional experiments were then performed to further examine the involvement of TRIM21 and proteasome activity in NS1 turnover.

Additional experiments were performed to examine the involvement of TRIM21 and the ubiquitin–proteasome system in NS1 turnover. Increasing the amount of Flag-TRIM21 plasmid from 0 to 1000 ng progressively reduced Myc-NS1 abundance, with normalized NS1/GAPDH values of 1.00, 0.70, 0.22, and 0.11, respectively. In the MG132 experiment, CCT2 expression modestly increased NS1 abundance under DMSO-treated conditions, whereas MG132 treatment resulted in greater NS1 accumulation. The highest NS1 level was detected when CCT2 expression and MG132 treatment were combined, with NS1/GAPDH values of 1.00, 1.12, 1.41, and 1.70 in the vector/DMSO, CCT2/DMSO, vector/MG132, and CCT2/MG132 groups, respectively (Figure 5F). Furthermore, anti-Myc immunoprecipitation showed that TRIM21 was recovered with NS1 (Figure 5G) and that high-molecular-weight HA-reactive ubiquitin conjugates associated with the NS1 immunocomplex became more prominent following TRIM21 expression and accumulated further after MG132 treatment (Figure 5H). These results support TRIM21-associated ubiquitination and proteasome-related turnover of NS1, together with a stabilizing effect associated with CCT2. However, because the immunoprecipitation was performed under non-denaturing conditions and neither a ligase-defective TRIM21 mutant nor pathway-epistasis analysis was included, the present data do not establish that TRIM21 directly ubiquitinates NS1 or that CCT2 stabilizes NS1 exclusively through the ubiquitin–proteasome pathway.

### 3.6. NS1 Expression Is Associated with Reduced CCT2 Recovery in LC3C Immunoprecipitates Under Overexpression Conditions, Together with Alterations in Autophagy-Related Markers

**Figure 6:**
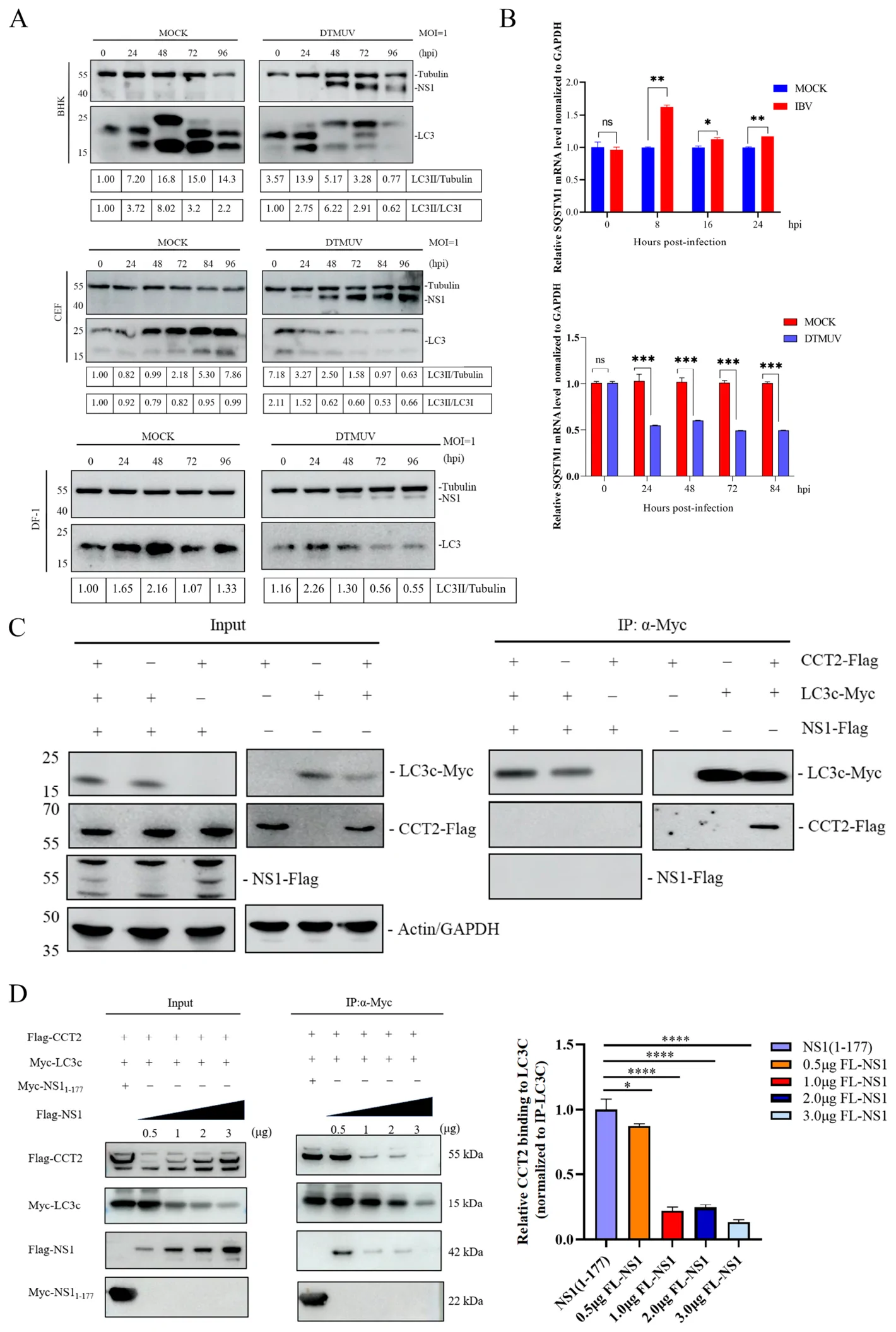
Immunoblot and co-immunoprecipitation assays for autophagy-related markers and CCT2-LC3C interaction. (**A**) Western blot analysis of LC3-II abundance in DTMUV-infected BHK-21, CEF, and DF-1 cells collected at the indicated hours post-infection (hpi). Tubulin served as the loading control, and LC3-II/tubulin ratios are shown. (**B**) RT-qPCR analysis of SQSTM1 mRNA expression in DTMUV-infected DF-1 cells relative to matched mock-infected controls. (**C**) Co-immunoprecipitation analysis of CCT2 recovery in LC3C immunoprecipitates in HEK293T cells expressing Myc-LC3C, Flag-CCT2, and Flag-NS1. (**D**) Dose-gradient co-immunoprecipitation analysis of CCT2 recovery in LC3C immunoprecipitates. The zero-NS1/empty-vector control is shown in panel (**C**). Data represent the mean ± SD from three independent biological replicates analyzed by one-way ANOVA.* *p* < 0.05; ** *p* < 0.01; *** *p* < 0.001; **** *p* < 0.0001; ns, not significant (*p* > 0.05).

To investigate if interaction of NS1 with CCT2 would play a regulatory role in this process during DTMUV infection and the underlying mechanism, we first evaluated the impact of DTMUV infection on key autophagy markers. In BHK-21, CEF, and DF-1 cells infected with DTMUV, reduced LC3-II protein abundance was observed at later time points (Figure 6A), consistent with altered autophagy-associated signaling during infection. To further characterize these marker changes, RT-qPCR was then used to assess the mRNA expression of another autophagy marker, SQSTM1/p62, in DF-1 cells infected with IBV and DTMUV, respectively. Compared with its matched mock-infected control, DTMUV infection was associated with reduced SQSTM1 expression (Figure 6B). The IBV-infected group was included only as an additional autophagy-related reference and was not used as the biological control for DTMUV. Because CCT2 has been reported to function as an aggrephagy receptor in other settings, we examined whether NS1 expression was associated with changes in CCT2–LC3C co-immunoprecipitation. The results showed CCT2 can indeed interact with LC3C (Figure 6C). However, CCT2 recovery in LC3C immunoprecipitates was reduced by co-transfection with NS1 (Figure 6C). These findings show that NS1 co-expression was associated with reduced CCT2 recovery in LC3C immunoprecipitates. In the dose-gradient Co-IP assay (Figure 6D), increasing full-length Flag-NS1 expression (0.5, 1, 2, and 3 μg) resulted in a dose-associated reduction in CCT2 recovery in LC3C immunoprecipitates. Importantly, immunoprecipitated Myc-LC3C bait also decreased across the NS1 dose gradient; The NS1_1–177_ truncation mutant did not produce this reduction.

## 4. Discussion

DTMUV NS1 protein, highly conserved among the family, represents one of the key determinants of disease severity [16]. As a multifunctional protein, NS1 participates in viral infection through multiple mechanisms, including forming viral replication complexes to facilitate RNA synthesis, interacting with complement components, disrupting endothelial permeability, and promoting viral invasion into host cells [17]. Although substantial research has been conducted regarding the roles of this protein in viral replication, immune evasion, pathogenesis, and interactions with natural hosts, the molecular mechanisms underlying these functions remain incompletely understood [18]. In the present study, IP-MS and co-immunoprecipitation identified CCT2 as an NS1-associated host factor, and the NS1 region required for formation of the NS1-CCT2 intracellular complex was mapped to residues 172–352. Functional assays demonstrated that CCT2 overexpression increased intracellular NS1 accumulation and elevated viral titers at late infection stages, whereas CCT2 knockdown delayed NS1 and E protein accumulation and reduced progeny virus production. Consistent with these findings, a recent study demonstrated that CCT2 knockdown similarly inhibits Zika virus replication [19]. In contrast, although CCT6 also interacted with NS1, CCT6 knockdown did not significantly alter NS1 mRNA or protein abundance under the tested conditions. These findings support a selective proviral role of CCT2 during DTMUV infection in the cell models used, while potential context-dependent or compensatory functions of CCT6 remain to be further investigated.

The NS1 region required for assembly of the NS1-CCT2 complex resides within the β-ladder domain of NS1 spanning amino acid residues 172–352, a domain composed of 18 β-strands. This domain may play a vital role in the viral replication process, possibly by promoting the assembly and structural stability of the viral replication complex [20]. It also mediates multiple immune-evasion processes, acting as a binding platform for complement-related proteins such as factor H, C1s, C4, and C4-binding protein to suppress complement activation [21]. Conserved C-terminal residues within this domain, particularly Pro320 and Met333, can markedly modulate antiviral signaling without altering viral replication fitness [22].

The interaction between flavivirus proteins and the host ubiquitin system is multifaceted. Host cells may utilize the ubiquitin system to mark viral proteins for degradation and regulate host factors to restrict viral replication. For instance, the host E3 ligase WWP2 interacts with Zika virus NS1, mediating K63-linked ubiquitination at K265 and K48-linked ubiquitination at K284 [8]. The latter modification marks NS1 as a proteasome degradation target, thereby inhibiting viral replication in mammalian systems. Conversely, during dengue virus infection, the deubiquitination of lipid droplet-associated protein Aup1 enhances its interaction with viral protein NS4A, leading to lipid droplet degradation to provide energy for viral replication [23]. The ubiquitination of Aup1 disrupts this interaction and inhibits viral replication. On the other hand, viruses have evolved sophisticated strategies to hijack or exploit the host ubiquitin system. For example, Zika virus infection triggers the activation of the NLRP3 inflammasome, and NS1 recruits the host deubiquitinase USP8 to remove the K11-linked ubiquitin chain from the K134 site of caspase-1, thereby inhibiting the proteasomal degradation of caspase-1, which in turn further enhances the activation of the NLRP3 inflammasome, ultimately benefiting viral replication. USP38 inhibits viral infection by removing K48- and K63-linked ubiquitin chains from the Zika virus E protein, and serves as a critical factor for the host resistance to this virus [24]. Our results showed that NS1 is modified by K48- and K63-linked polyubiquitination. CHX-chase assays, TRIM21 dose-gradient treatments, MG132 inhibition, and ubiquitin detection experiments collectively indicate that NS1 abundance is regulated via the proteasome pathway, consistent with a CCT2-dependent stabilizing effect on intracellular NS1. Nevertheless, the specific ubiquitin-acceptor lysine residues of DTMUV NS1 remain uncharacterized. Current data cannot confirm direct TRIM21-mediated NS1 ubiquitination, nor do they exclude CCT2 functions independent of the ubiquitin–proteasome system. Thus, additional CCT2-associated mechanisms, including modulation of NS1 folding, trafficking, synthesis, or alternative degradation pathways, may also contribute to NS1 regulation. Zika virus NS2A undergoes ubiquitination through the host E3 ubiquitin ligase AMFR. Ubiquitinated NS2A interacts with the key ER autophagy receptor FAM134B and induces its degradation, thereby inhibiting protective ER autophagy and ultimately enhancing viral pathogenicity [25]. Notably, this AMFR-mediated NS2A ubiquitination and its interference with FAM134B are highly conserved in various flaviviruses, including dengue virus, West Nile virus, and Japanese encephalitis virus [26]. CCT2 acts as an aggrephagy receptor through interactions with ATG8-family proteins [5]. DTMUV infection alters autophagy-related molecular markers such as LC3-II and SQSTM1 mRNA, yet these steady-state readouts alone cannot prove that disrupted CCT2-LC3C complex formation leads to impaired aggrephagy or aggregate-clearance defects. Under overexpression conditions, DTMUV NS1 dose-dependently reduces CCT2 recovery in the CCT2-LC3C complex, an effect absent for the NS1_1–177_ mutant; the present data only support dose-dependent reduction in CCT2 recovery and do not validate a binding capacity-dependent mechanism.

In summary, the present study demonstrates the physical interaction between DTMUV NS1 protein and CCT2. CCT2 acts as a proviral host factor for DTMUV: it stabilizes intracellular NS1 protein and supports the production of infectious viral progeny. Several limitations of this study should be noted. The endogenous Co-IP experiment lacked standard IgG isotype controls, and all functional experiments were performed in immortalized cell lines. Furthermore, replication-competent NS1 mutants with specific defects in CCT2-binding are currently unavailable for functional validation. Future investigations, including optimized control experiments for endogenous protein–protein interaction, autophagic-flux and aggregate-clearance assays, CCT2-binding-deficient viral mutants, primary duck cells, and infected duck tissues, are required to dissect the physiological relevance of the NS1-CCT2 axis. Collectively, these findings expand our understanding of flavivirus-host protein–protein interaction mechanisms. Targeting the NS1-CCT2 protein-protein interface may represent a promising strategy for developing anti-DTMUV interventions. Nevertheless, further studies are still needed to elucidate the physiological significance of the NS1-CCT2-LC3C axis in virus-infected primary duck cells and animal tissues.

## Data Availability

The original contributions presented in this study are included in the article/Supplementary Material. Further inquiries can be directed to the corresponding authors.

## Supplementary Materials

The following supporting information can be downloaded at: https://www.mdpi.com/article/10.3390/vetsci13090979/s1, File S1: Original images of Western-blotting.

## Appendix A. Primer Information

**Table A1:** Primers used for construction of plasmids.

Primers	Sequence (5′→3′)
pXJ40-Flag-F	acatcctggtcatcatcctg
pXJ40-Flag-R	accacaactagaatgcagtg
pXJ40-Myc-F	atcctgcctttctctttatggt
pXJ40-Myc-R	taaccgtattaccgcctttgagtg
pCMV-Myc-F	tctaaaagctgcggaattgt
pCMV-Myc-R	cagcttataatggttacaaat
pCMV-Flag-F	tctaaaagctgcggaattgt
pCMV-Flag-R	cagcttataatggttacaaat
NS1-F	gaattccggacacggggtgctcaat
NS1-R	ggggtaccagccatgacctttgatt
CCT2-F	gaattccgatggcgtccctttccc
CCT2-R	ccgctcgagttaacaggggtggtgatcag
CCT1-F	aaggaaaaaagcggccgcatggaggggccttt
CCT1-R	ggggtacctcaatcattaagggctccag
CCT3-F	cccaagcttatgatgggccatcgt
CCT3-R	ggggtaccttgcctagcactcact
CCT4-F	ccgctcgagatgcccgagaatgtgg
CCT4-R	cggggtaccttatcgagtgtttaccacatcatc
CCT5-F	acatggtacaccatggcgtccat
CCT5-R	acatgcatgcgcatcacgagacatccat
CCT6-F	acatgcatgcatggcggcggtgaa
CCT6-R	cggggtacctcaacctttcagagaagacatt
CCT7-F	gaattccgatgatgcccacacca
CCT7-R	ggggtaccagccatgtgatgggt
CCT8-F	gaattccgatggcgcttcacgtt
CCT8-R	ggggtaccccaatttcaatcattttggtc
NS1(1–177)-F	gaattccggacacggggtgctcaa
NS1(1–177)-R	gggtacctcaagttgtattttctgttcgc
NS1(172–352)-F	cgggatcccgaacagaaaatacaactgattg
NS1(172–352)-R	ggggtacctcaagccatgacctttgattt

All gene sequences in the provided tables are denoted in lowercase letters. For plasmid construction, the corresponding vector sequences were incorporated upstream of target genes during PCR amplification, followed by cloning via either homologous recombination or T4 DNA ligase-mediated ligation.

**Table A2:** Primer sequences for RT-qPCR.

Primers	Sequence (5′-3′)
q-GAPDH-F	gcaccaccaactgcttagcc
q-GAPDH-R	ccatcacgccacagctttcc
q-CCT2-F	tggagaagacaaactcattcac
q-CCT2-R	aagggcatcatgcaaagac
qDTMUV-NS1-F	cgggatccatggacacggggtgctcaatc
qDTMUV-NS1-R	cccaagctttcaagccatgacctttgatttg
SQSTM1-F	TCATTCACTCCCTCTCCCAGATGC
SQSTM1-R	CCGCTCCCATCTCATACTTCTTGC
