# Supplementary material for: NS1 of Duck Tembusu Virus Interacts with CCT2: Reduced CCT2 Recovery in LC3C Co-Immunoprecipitation In Vitro and the Proviral Role of CCT2 in DTMUV Infection
Source: Vet Sci. 2026 Sep 17;13(9):979. doi: 10.3390/vetsci13090979 (PMC13612084; doi:10.3390/vetsci13090979)
Supplement: Supplementary file 1 [file vetsci-13-00979-s001.zip › vetsci-4433183-supplementary.pdf]

Supplementary Materials

Figure S2B

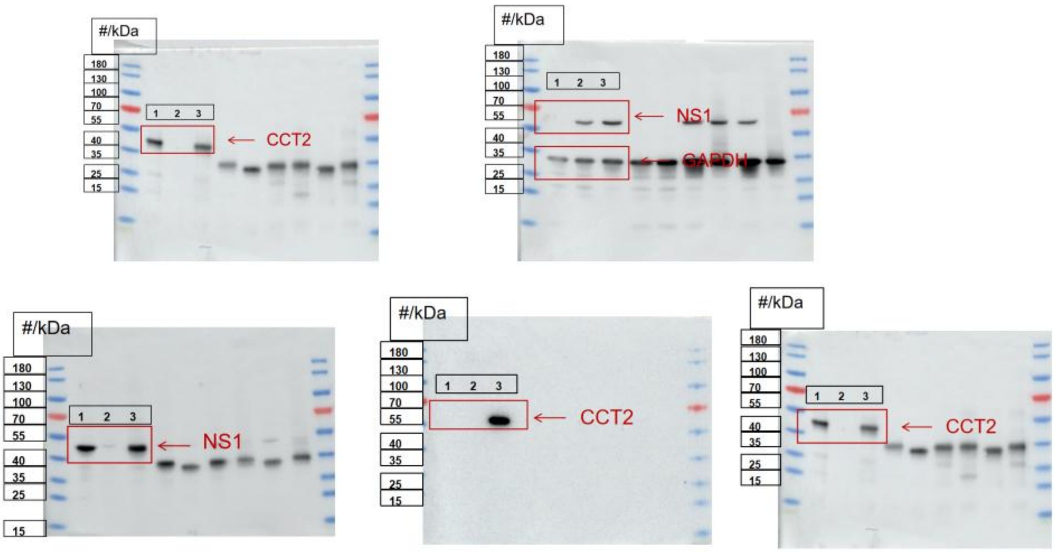

Figure S2B： Co‑immunoprecipitation assay to verify the interaction between NS1 and CCT2. Western blot membranes of NS1-Myc (~42 kDa), CCT2-Flag (~60 kDa), and GAPDH (~36 kDa) proteins were detected with anti‑ Myc (1:2000; Nulen Biotech, China), anti‑ Flag (1:2000; Nulen Biotech, China), and anti‑ GAPDH (1:2000; Proteintech, China) antibodies. Gel‑ separated proteins were transferred to nitrocellulose membranes (Merck Millipore, USA). Membranes were incubated with horseradish peroxidase‑ conjugated goat anti‑ mouse/anti‑ rabbit IgG secondary antibody (1:5000), and bands were visualized using ECL chemiluminescent substrate (Beyotime, China). #Weight marker (molecular weight in kDa): Thermo Scientific™ PageRuler™ Prestained Protein Ladder, 10 to 180 kDa; catalogue number: 26616. Blot images, prior to densitometry readings, were converted to grayscale with ImageJ (ImageJ v.1.49, National Institutes of Health, Maryland, USA) as follows: Image → Type → 8 bit, next: Image → Adjust → Brightness/Contrast → Auto. Input: whole-cell lysates before immunoprecipitation; IP: α‑ Myc: protein complexes immunoprecipitated using anti‑ Myc antibody; +, plasmid transfection was performed; −, plasmid transfection was omitted.

Figure S2C

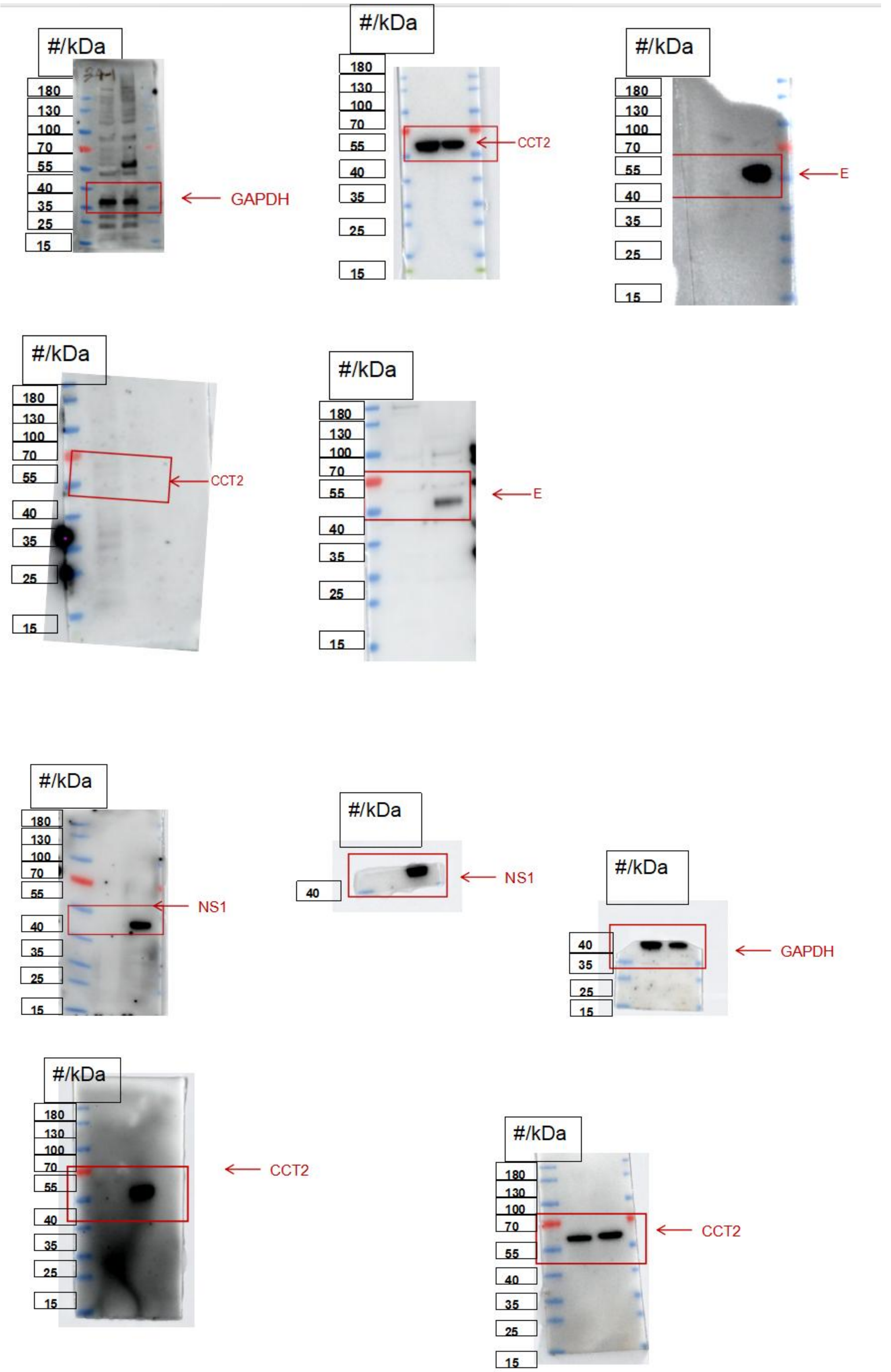

Figure S2C:Co- immunoprecipitation assay to verify the interaction between endogenous

CCT2 and viral E or NS1 protein during DTMUV infection. Western blot membranes of E (~42 kDa), NS1 (~42 kDa), CCT2 (~60 kDa), and GAPDH (~36 kDa) proteins were detected with anti-E monoclonal antibody (1:5000; generated in-house in our laboratory), anti-NS1 monoclonal antibody (1:5000; generated in-house in our laboratory), anti-CCT2 (1:2000; Proteintech, China), and anti-GAPDH (1:2000; Proteintech, China) antibodies. Gel-separated proteins were transferred to nitrocellulose membranes (Merck Millipore, USA). Membranes were incubated with horseradish peroxidase-conjugated goat anti-mouse/anti-rabbit IgG secondary antibody (1:5000), and bands were visualized using ECL chemiluminescent substrate (Beyotime, China). #Weight marker (molecular weight in kDa): Thermo Scientific™ PageRuler™ Prestained Protein Ladder, 10 to 180 kDa; catalogue number: 26616. Blot images, prior to densitometry readings, were converted to grayscale with ImageJ (ImageJ v.1.49, National Institutes of Health, Maryland, USA) as follows: Image → Type → 8 bit, next: Image → Adjust → Brightness/Contrast → Auto. Input: whole-cell lysates before immunoprecipitation; IP: α-E / IP: α-NS1: protein complexes immunoprecipitated using anti-E or anti-NS1 antibody; +, DTMUV infection was performed; −, DTMUV infection was omitted.

FigureS2 D

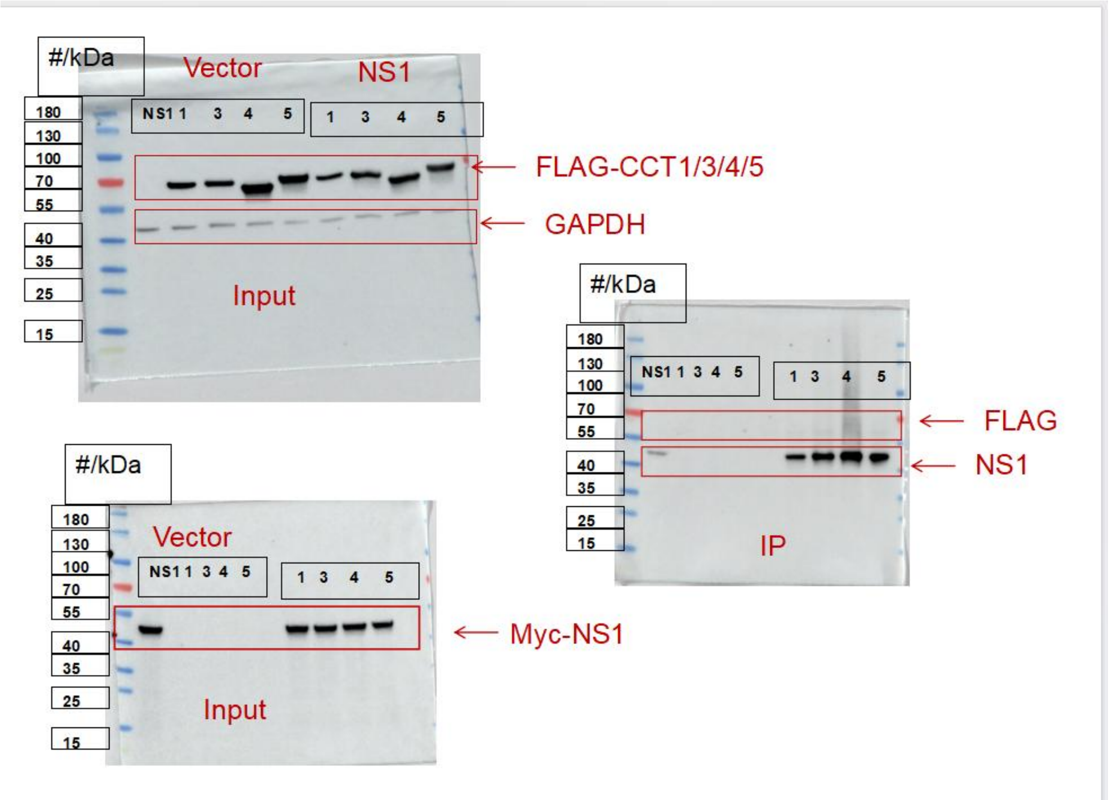

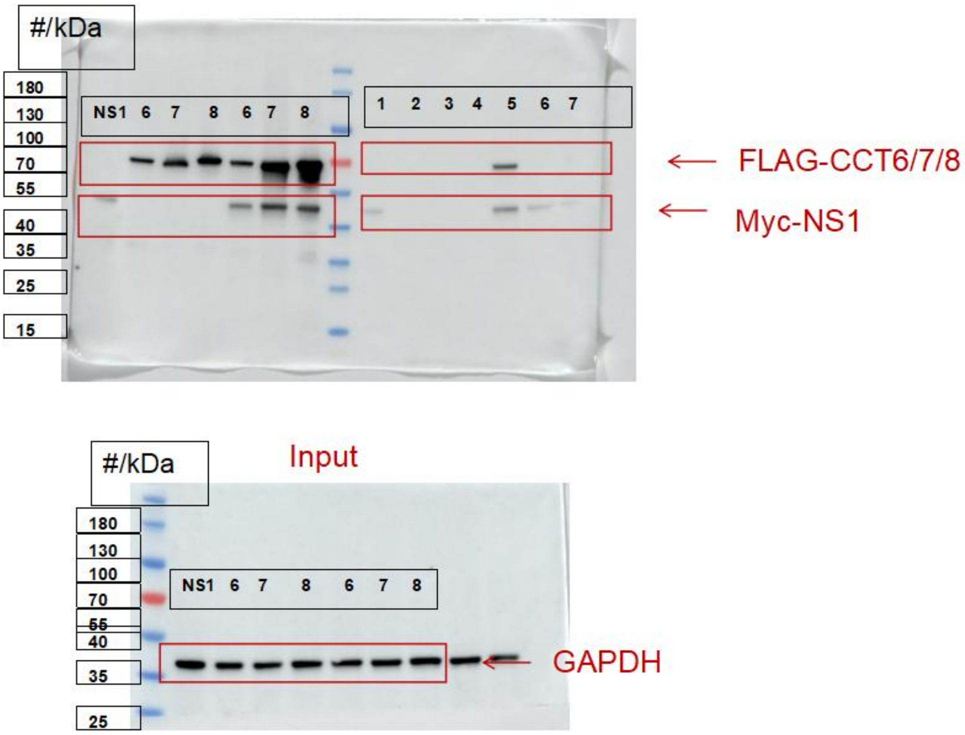

Figure S2D: Co- immunoprecipitation assay screening for the interaction between NS1 and different CCT isoforms (CCT2, CCT3, CCT4, CCT5, CCT6, CCT7, CCT8). Western blot membranes of NS1-Myc (~42 kDa), CCT2-Flag / CCT3-Flag / CCT4-Flag / CCT5-Flag / CCT6-Flag / CCT7-Flag / CCT8-Flag (~55–60 kDa), and GAPDH (~36 kDa) proteins were detected with anti-Myc (1:2000; Nulen Biotech, China), anti-Flag (1:2000; Nulen Biotech, China), and anti-GAPDH (1:2000; Proteintech, China) antibodies. Gel-separated proteins were transferred to nitrocellulose membranes (Merck Millipore, USA). Membranes were incubated with horseradish peroxidase-conjugated goat anti-mouse/anti-rabbit IgG secondary antibody (1:5000), and bands were visualized using ECL chemiluminescent substrate (Beyotime, China). #Weight marker (molecular weight in kDa): Thermo Scientific™ PageRuler™ Prestained Protein Ladder, 10 to 180 kDa; catalogue number: 26616. Blot images, prior to densitometry readings, were converted to grayscale with ImageJ (ImageJ v.1.49, National Institutes of Health, Maryland, USA) as follows: Image → Type → 8 bit, next: Image → Adjust → Brightness/Contrast → Auto. Input: whole-cell lysates before immunoprecipitation; IP: α-Myc: protein complexes immunoprecipitated using anti-Myc antibody. Vector: empty plasmid control group; NS1: cells transfected with NS1-Myc plasmid together with different CCT-Flag plasmids.

Figure S2E

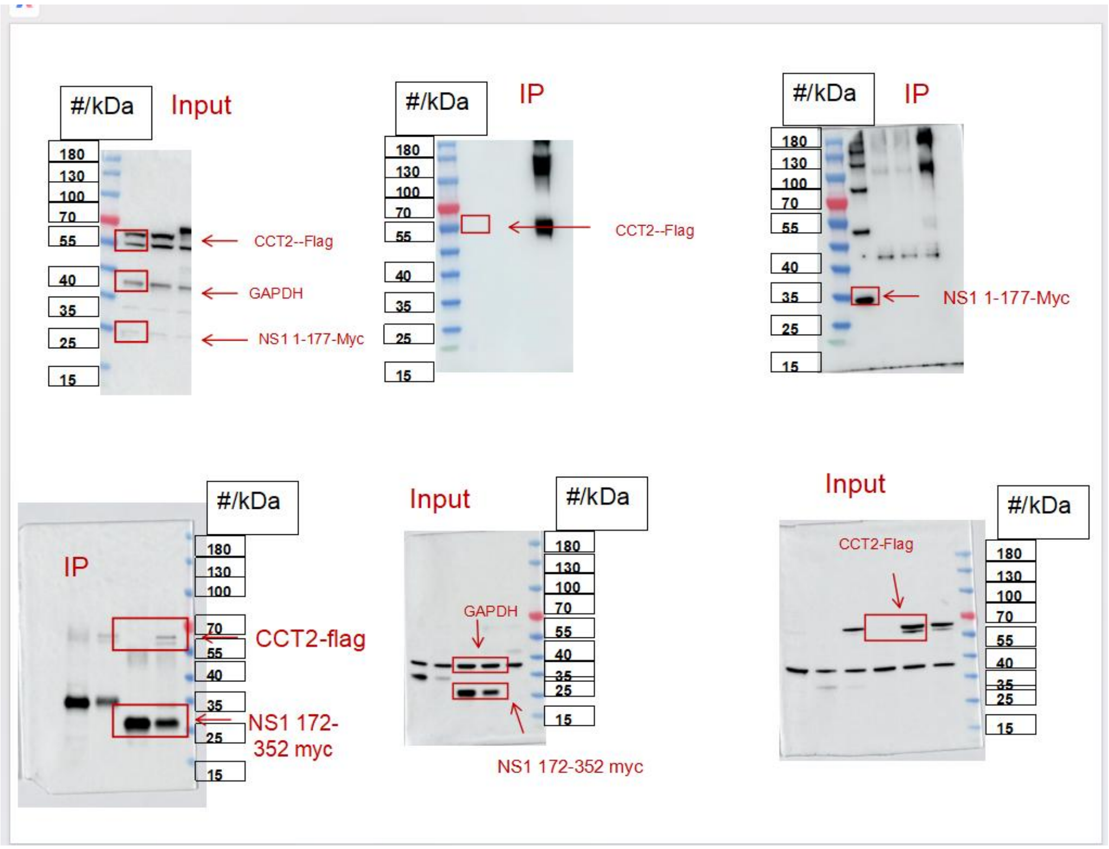

Figure S2E： Figure S2E. Co- immunoprecipitation assay identifying the NS1 domain responsible for binding to CCT2. Western blot membranes of CCT2-Flag (~60 kDa), $NS1_{1-177}$-Myc (~22 kDa), $NS1_{172-352}$-Myc (~20 kDa), and GAPDH (~36 kDa) proteins were detected with anti-Myc (1:2000; Nulen Biotech, China), anti-Flag (1:2000; Nulen Biotech, China), and anti-GAPDH (1:2000; Proteintech, China) antibodies. Gel-separated proteins were transferred to nitrocellulose membranes (Merck Millipore, USA). Membranes were incubated with horseradish peroxidase-conjugated goat anti-mouse/anti-rabbit IgG secondary antibody (1:5000), and bands were visualized using ECL chemiluminescent substrate (Beyotime, China). #Weight marker (molecular weight in kDa): Thermo Scientific™ PageRuler™ Prestained Protein Ladder, 10 to 180 kDa; catalogue number: 26616. Blot images, prior to densitometry readings, were converted to grayscale with ImageJ (ImageJ v.1.49, National Institutes of Health, Maryland, USA) as follows: Image → Type → 8 bit, next: Image → Adjust → Brightness/Contrast → Auto. Input: whole-cell lysates before immunoprecipitation; IP: α-Myc: protein complexes immunoprecipitated using anti-Myc antibody; +, plasmid transfection was performed; −, plasmid transfection was omitted.

Figure S3B

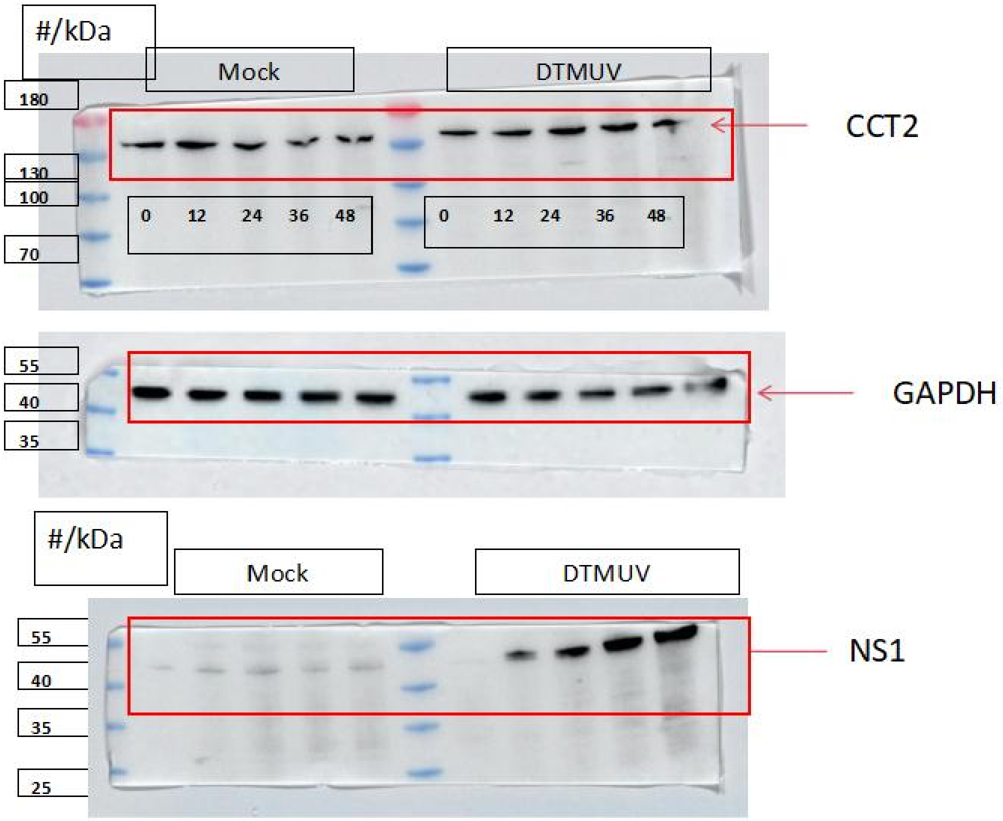

Figure S3B： Western blot analysis of endogenous CCT2 protein expression at different time points after DTMUV infection (MOI = 1). Western blot membranes of CCT2 (~60 kDa), NS1 (~42 kDa), and GAPDH (~36 kDa) proteins were detected with anti-CCT2 (1:2000; Proteintech, China), anti-NS1 monoclonal antibody (1:5000; generated in-house in our laboratory), and anti-GAPDH (1:2000; Proteintech, China) antibodies. Gel-separated proteins were transferred to nitrocellulose membranes (Merck Millipore, USA). Membranes were incubated with horseradish peroxidase-conjugated goat anti-mouse/anti-rabbit IgG secondary antibody (1:5000), and bands were visualized using ECL chemiluminescent substrate (Beyotime, China). #Weight marker (molecular weight in kDa): Thermo Scientific™ PageRuler™ Prestained Protein Ladder, 10 to 180 kDa; catalogue number: 26616. Blot images, prior to densitometry readings, were converted to grayscale with ImageJ (ImageJ v.1.49, National Institutes of Health, Maryland, USA) as follows: Image → Type → 8 bit, next: Image → Adjust → Brightness/Contrast → Auto. MOCK: uninfected control group; DTMUV: duck Tembusu virus infected group; hpi: hours post infection. The values below the bands represent the relative gray values of CCT2 normalized to GAPDH.

Figure S3C

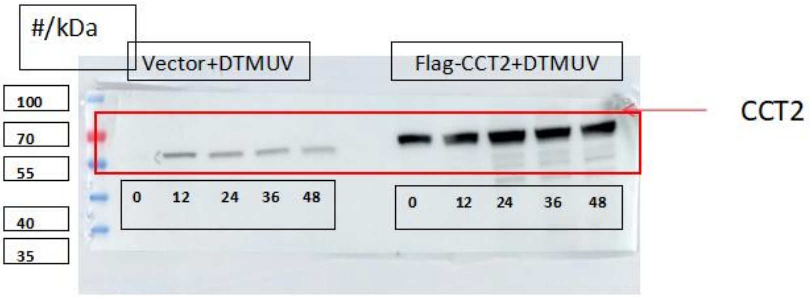

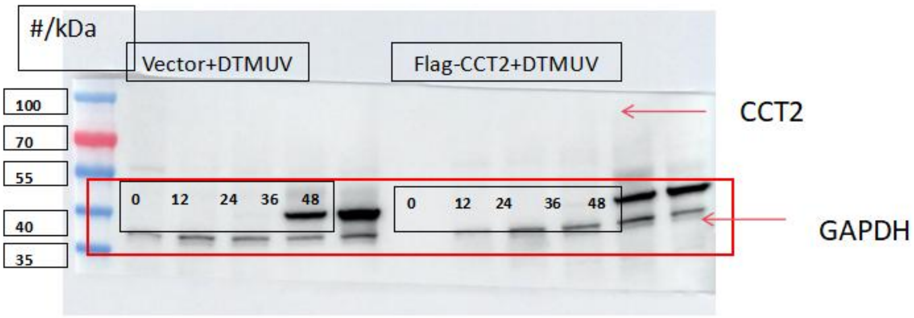

Figure S3C: Western blot analysis of NS1 protein expression following CCT2 overexpression during DTMUV infection (MOI = 1). Western blot membranes of CCT2 (~60 kDa), NS1 (~42 kDa), and GAPDH (~36 kDa) proteins were detected with anti- CCT2 (1:2000; Proteintech, China), anti- NS1 monoclonal antibody (1:5000; generated in- house in our laboratory), and anti- GAPDH (1:2000; Proteintech, China) antibodies. Gel- separated proteins were transferred to nitrocellulose membranes (Merck Millipore, USA). Membranes were incubated with horseradish peroxidase- conjugated goat anti- mouse/anti- rabbit IgG secondary antibody (1:5000), and bands were visualized using ECL chemiluminescent substrate (Beyotime, China). #Weight marker (molecular weight in kDa): Thermo Scientific™ PageRuler™ Prestained Protein Ladder, 10 to 180 kDa; catalogue number: 26616. Blot images, prior to densitometry readings, were converted to grayscale with ImageJ (ImageJ v.1.49, National Institutes of Health, Maryland, USA) as follows: Image → Type → 8 bit, next: Image → Adjust → Brightness/Contrast → Auto. Vector: empty plasmid control group; CCT2: CCT2 overexpression group; hpi: hours post infection. The values below the bands represent the relative gray values of NS1 normalized to GAPDH.

Figure S4A

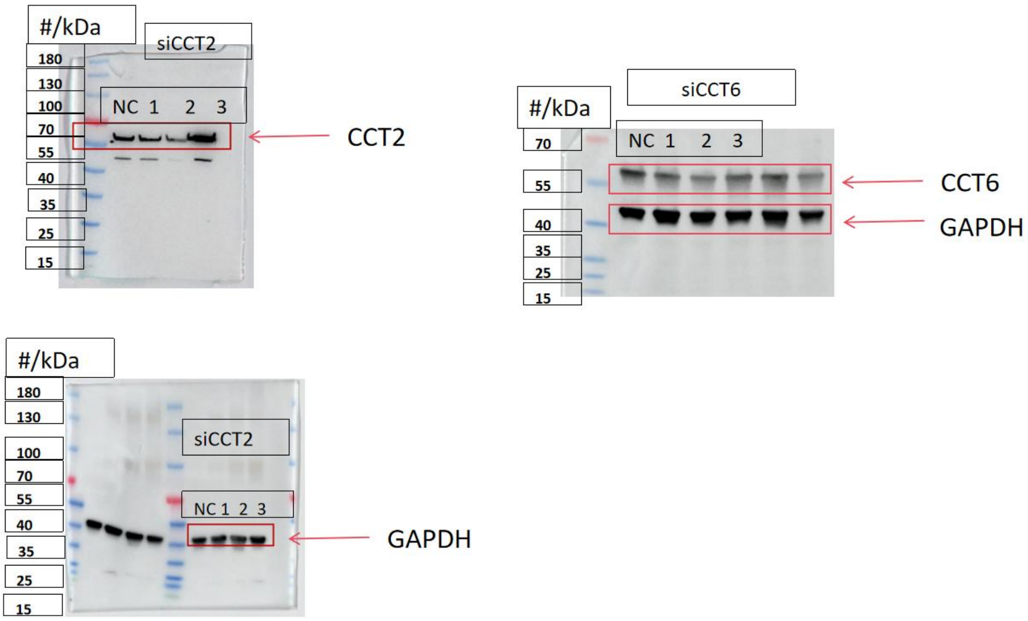

Figure S4A： Western blot analysis of the knockdown efficiency of siRNAs targeting CCT2 and CCT6. Western blot membranes of CCT2 (~60 kDa), CCT6 (~58 kDa) and GAPDH (~36 kDa) proteins were detected with anti-CCT2 (1:2000; Proteintech, China), anti-CCT6 (1:2000; Proteintech, China), and anti-GAPDH (1:2000; Proteintech, China) antibodies. Gel-separated proteins were transferred to nitrocellulose membranes (Merck Millipore, USA). Membranes were incubated with horseradish peroxidase-conjugated goat anti-mouse/anti-rabbit IgG secondary antibody (1:5000), and bands were visualized using ECL chemiluminescent substrate (Beyotime, China). #Weight marker (molecular weight in kDa): Thermo Scientific™ PageRuler™ Prestained Protein Ladder, 10 to 180 kDa; catalogue number: 26616. Blot images, prior to densitometry readings, were converted to grayscale with ImageJ (ImageJ v.1.49, National Institutes of Health, Maryland, USA) as follows: Image → Type → 8 bit, next: Image → Adjust → Brightness/Contrast → Auto. NC – negative control siRNA; 1, 2, 3 – three independent siRNA fragments targeting CCT2 or CCT6.

Figure S4D

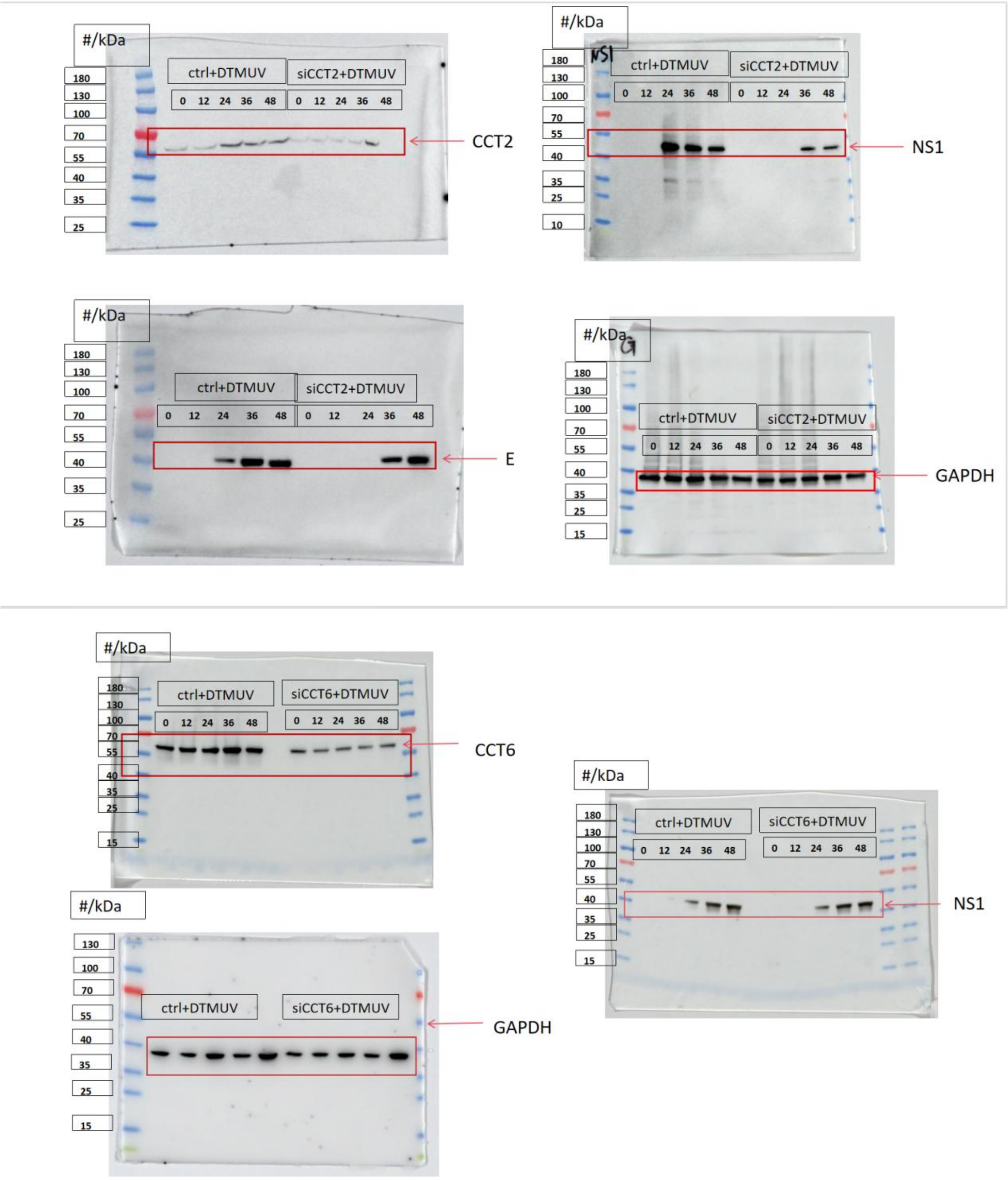

Figure S4D: Western blot detection of the effects of CCT2 or CCT6 knockdown on the expression of DTMUV NS1 and E proteins at different time points after virus infection. Western blot membranes of CCT2 (~60 kDa), CCT6 (~58 kDa), NS1 (~45 kDa), E (~42 kDa) and GAPDH (~36 kDa) proteins were detected with anti-CCT2 (1:2000; Proteintech, China), anti-CCT6 (1:2000; Proteintech, China), anti-NS1 monoclonal antibody (1:5000; generated in-house in our laboratory), anti-E monoclonal antibody (1:5000; generated in-house in our laboratory), and anti-GAPDH (1:2000; Proteintech, China) antibodies. Gel-separated proteins were transferred to nitrocellulose membranes (Merck Millipore, USA). Membranes were incubated with horseradish peroxidase-conjugated goat anti- mouse/anti-rabbit IgG secondary antibody (1:5000), and bands were visualized using ECL chemiluminescent substrate (Beyotime, China). #Weight marker (molecular weight in kDa):

Thermo Scientific™ PageRuler™ Prestained Protein Ladder, 10 to 180 kDa; catalogue number: 26616. Blot images, prior to densitometry readings, were converted to grayscale with ImageJ (ImageJ v.1.49, National Institutes of Health, Maryland, USA) as follows: Image → Type → 8 bit, next: Image → Adjust → Brightness/Contrast → Auto. ctrl – negative control siRNA; siCCT2 / siCCT6 – siRNA targeting CCT2 or CCT6; 0, 12, 24, 36, 48 – hours post DTMUV infection.

Figure S5A

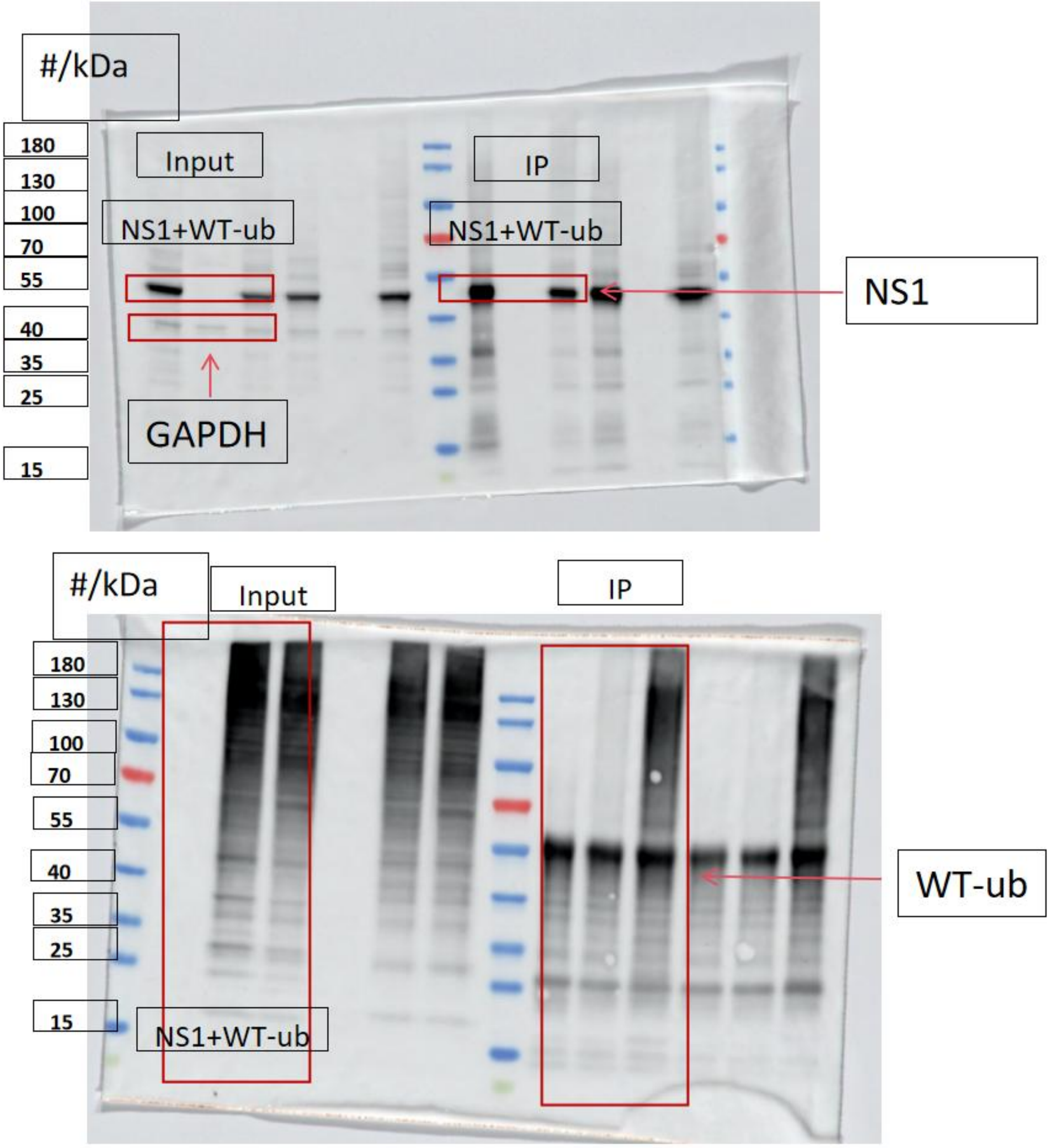

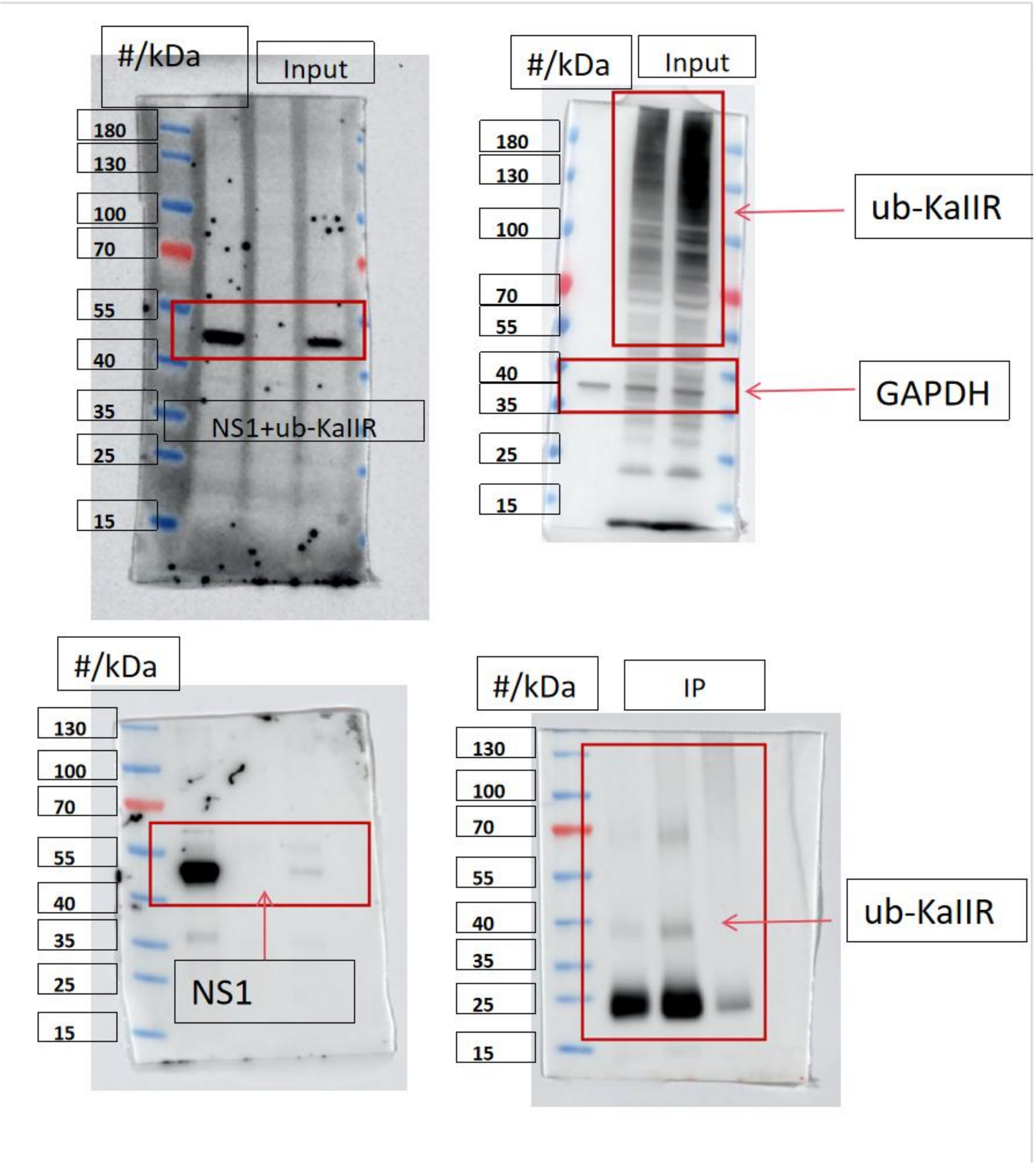
#/kDa
Input
180
130
100
70
55
40
35
25
15
NS1+ub-KaIIR
#/kDa
Input
180
130
100
70
55
40
35
25
15
ub-KaIIR
GAPDH
#/kDa
130
100
70
55
40
35
25
15
NS1
#/kDa
IP
130
100
70
55
40
35
25
15
ub-KaIIR

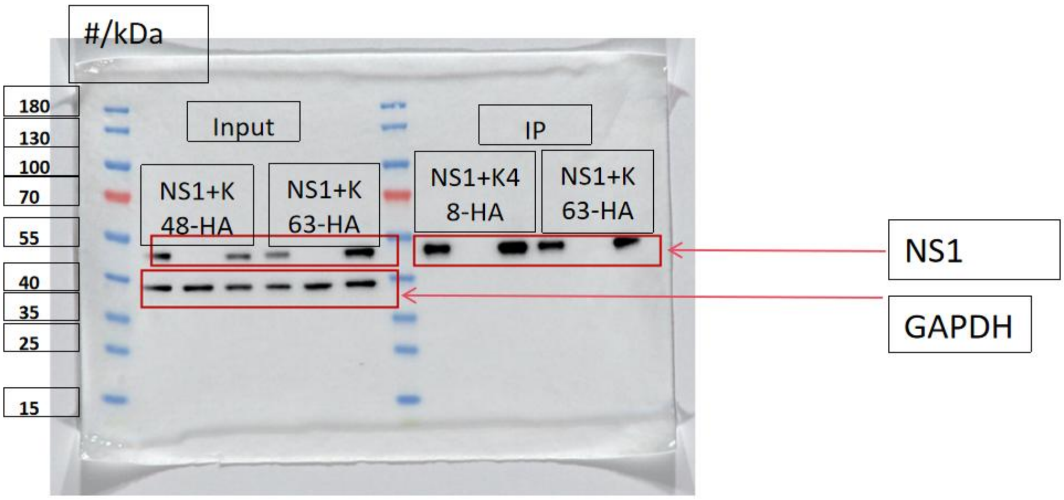

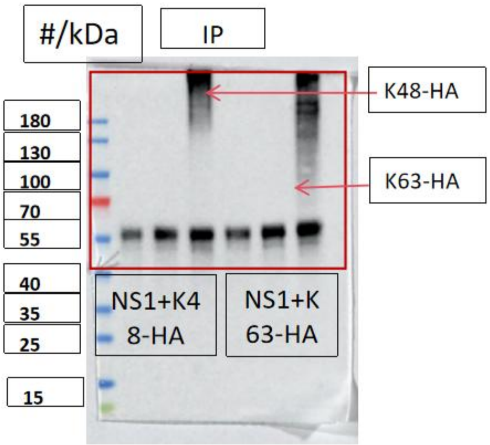

Figure S5A: Western blot membranes of NS1 (~42 kDa), GAPDH (~36 kDa), and HA-tagged ubiquitin variants (WT - Ub, ub - K48, ub - K63, ub - KallR). Anti - NS1 monoclonal antibody (1:5000; generated in - house in our laboratory), anti - GAPDH (1:2000; Proteintech, China), and anti- HA tag antibody (1:5000; Nulen Biotech, China) were used for immunoblotting. Gel - separated proteins were transferred to nitrocellulose membranes (Merck Millipore, USA). Membranes were incubated with horseradish peroxidase- conjugated goat anti- mouse/anti- rabbit IgG secondary antibody (1:5000), and bands were visualized using ECL chemiluminescent substrate (Beyotime, China). #Weight marker (molecular weight in kDa): Thermo Scientific™ PageRuler™ Prestained Protein Ladder, 10 to 180 kDa; catalogue number: 26616. Blot images, prior to densitometry readings, were converted to grayscale with ImageJ (ImageJ v.1.49, National Institutes of Health, Maryland, USA) as follows: Image → Type → 8 bit, next: Image → Adjust → Brightness/Contrast → Auto. Input – whole- cell lysate before immunoprecipitation; IP – protein complexes enriched by anti- Myc immunoprecipitation. WT- ub, ub- K48, ub - K63, ub- KallR – cells co- transfected with pCMV- myc- NS1 and wild- type ubiquitin, K48- only ubiquitin mutant, K63- only ubiquitin mutant or K- all- R ubiquitin mutant plasmids, respectively. This co - immunoprecipitation assay was performed to investigate the interaction between NS1 and different ubiquitin plasmid-

encoded products, and it only detects the intrinsic ubiquitination pattern of NS1 and does not involve CCT2.

Figure S5B

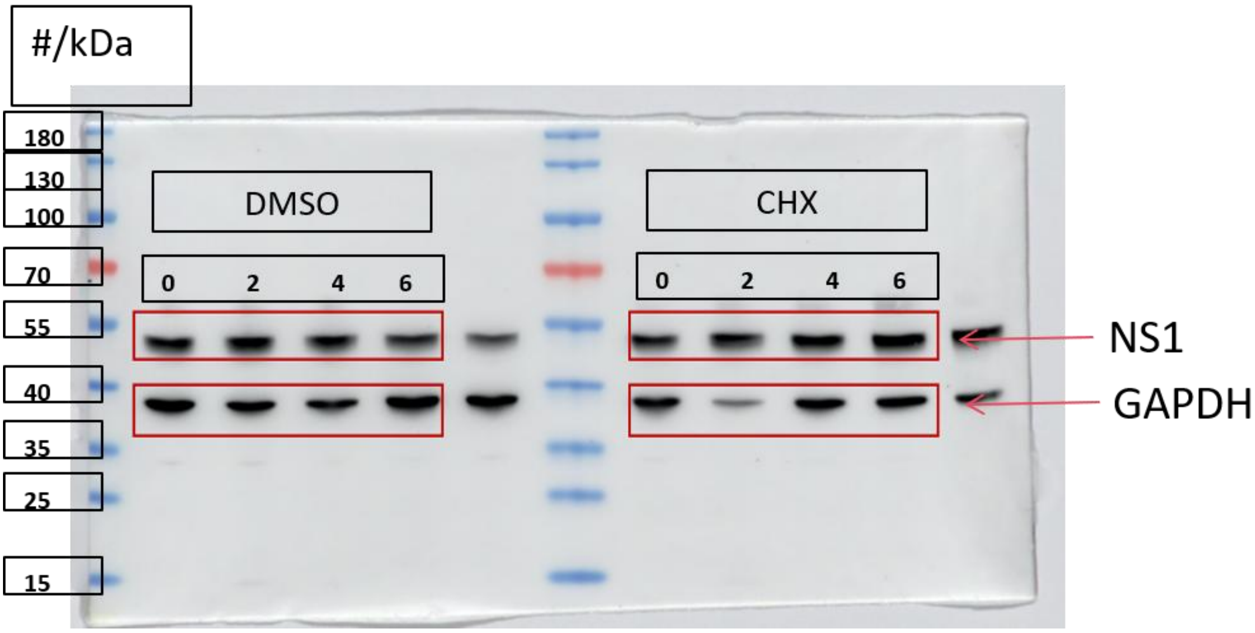

Figure S5B: Western blot membrane of NS1 (~42 kDa) and GAPDH (~36 kDa) proteins detected with anti-NS1 (1:2000; Proteintech, China) and anti-GAPDH (1:2000; Proteintech, China) antibodies. Gel-separated proteins were transferred to nitrocellulose membranes (0.2 μm pore size; Bio-Rad, Hercules, USA) by semi-dry electroblotting (1.5 mA per cm^2, 20 min). Membranes were incubated with a horseradish peroxidase-conjugated secondary antibody (AS09 602; 1:10000; Agrisera) and developed with Pierce™ DAB Substrate Kit (Thermo Fisher Scientific). Weight marker: Thermo Scientific™ PageRuler™ Prestained Protein Ladder, 10 to 180 kDa; catalog no. 26616. Blot images, prior to densitometry readings, were converted to grayscale with ImageJ (ImageJ v.1.49, National Institutes of Health, Maryland, USA) as follows: Image → Type → 8 bit, next: Image → Adjust → Brightness/Contrast → Auto. DMSO — cells treated with DMSO (vehicle control); CHX — cells treated with cycloheximide (CHX; [100 μg/mL]); 0, 2, 4, 6 — cells harvested at indicated hours post treatment.

Figure S5C

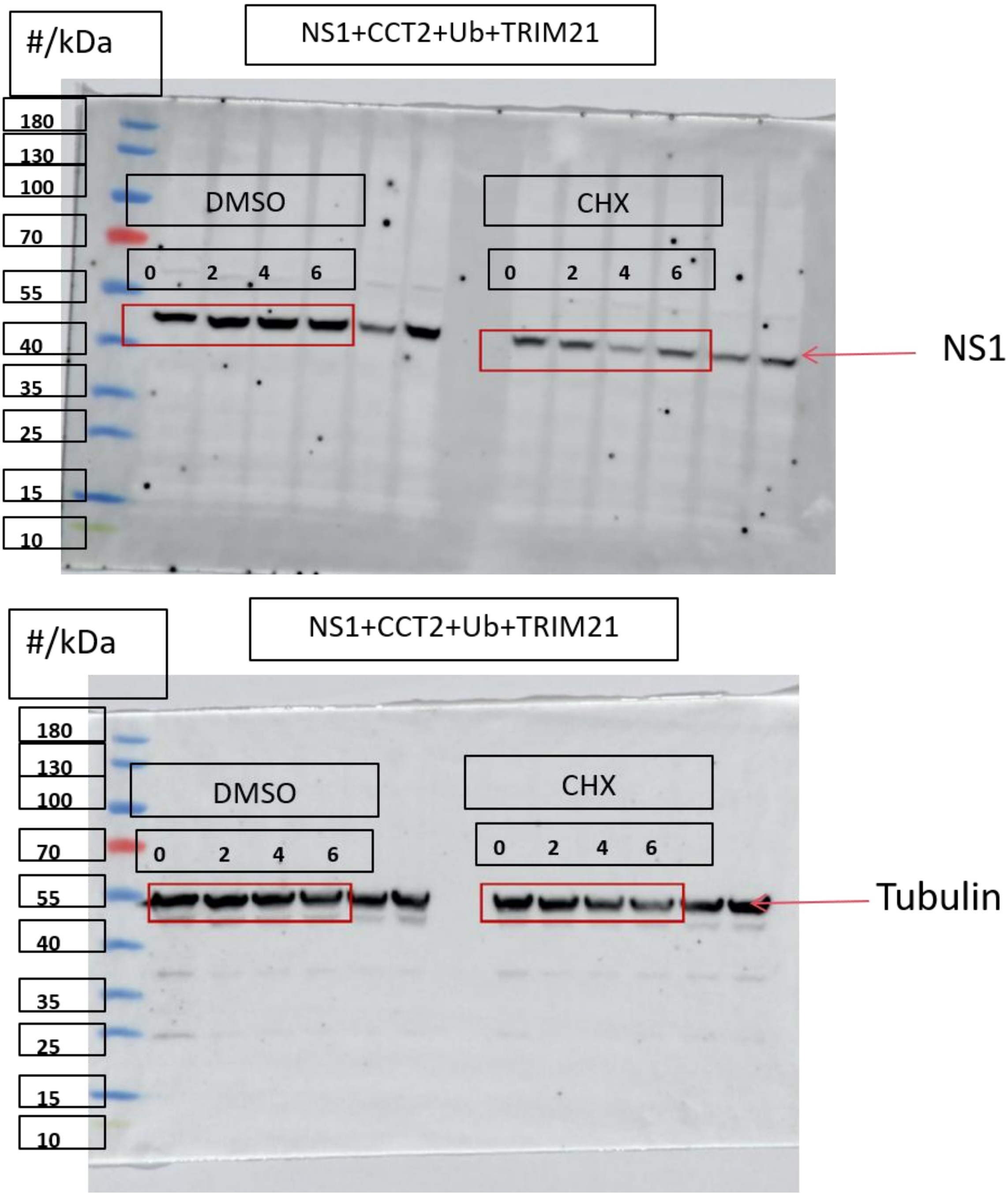

Figure S5C: Western blot membrane of NS1 (~42 kDa) and Tubulin (~55 kDa) proteins detected with anti-NS1 (1:2000; Proteintech, China) and anti-Tubulin (1:2000; Proteintech, China) antibodies. Gel-separated proteins were transferred to nitrocellulose membranes (0.2 μm pore size; Bio-Rad, Hercules, USA) by semi-dry electroblotting (1.5 mA per cm², 20 min). Membranes were incubated with a horseradish peroxidase-conjugated secondary antibody (AS09 602; 1:10000; Agrisera) and developed with Pierce™ DAB Substrate Kit (Thermo Fisher Scientific). Weight marker: Thermo Scientific™ PageRuler™ Prestained

Protein Ladder, 10 to 180 kDa; catalog no. 26616. Blot images, prior to densitometry readings, were converted to grayscale with ImageJ (ImageJ v.1.49, National Institutes of Health, Maryland, USA) as follows: Image → Type → 8 bit, next: Image → Adjust → Brightness/Contrast → Auto. Cells were co-transfected with NS1, CCT2, ubiquitin (Ub), and Trim21 expression plasmids, followed by treatment with DMSO (vehicle control) or cycloheximide (CHX; [100 µg/mL]). DMSO — cells treated with DMSO; CHX — cells treated with CHX; 0, 2, 4, 6 — cells harvested at indicated hours post treatment. Tubulin was used as a loading control.

Figure S5D

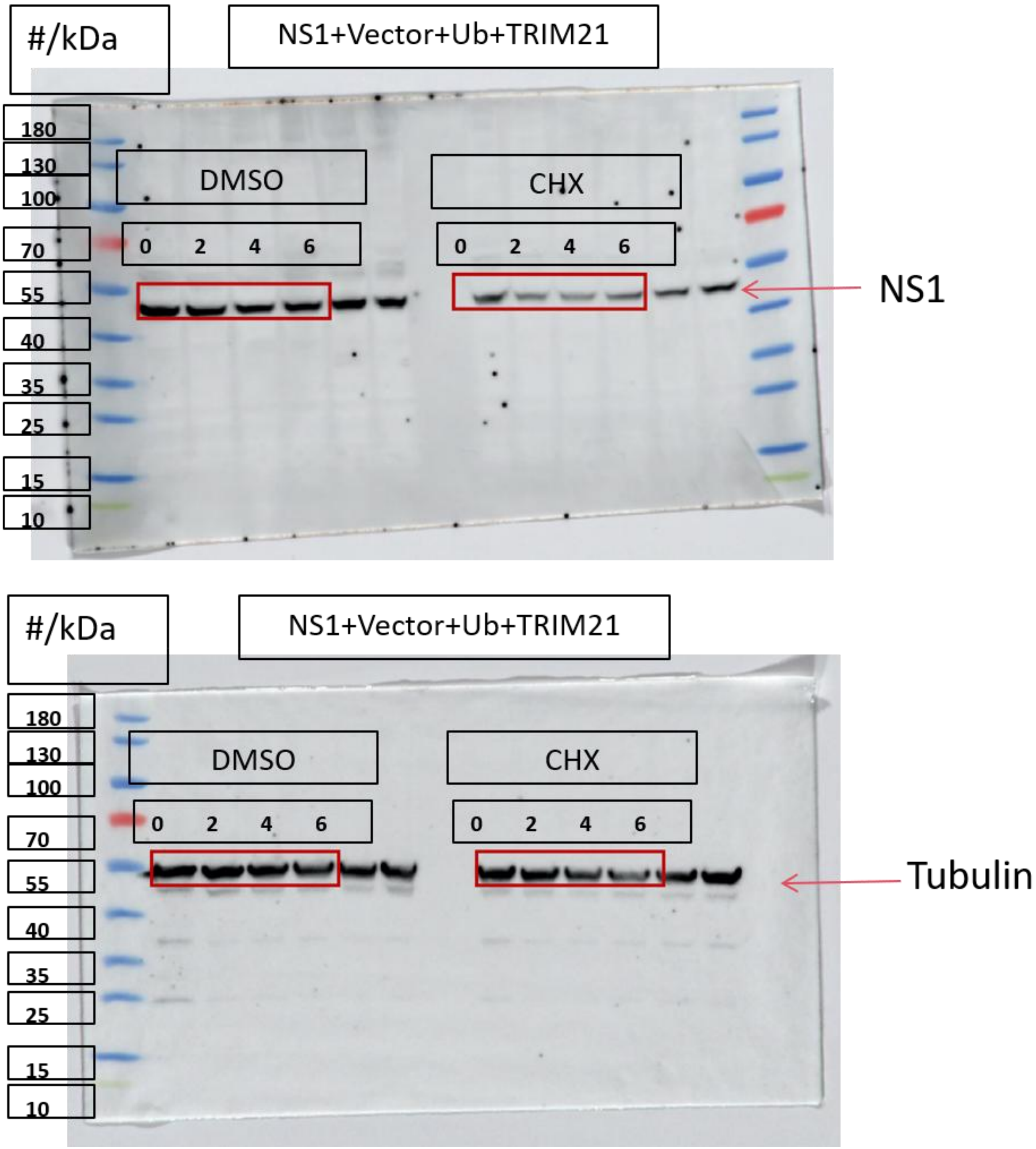

Figure S5D: Western blot membrane of NS1 (~42 kDa) and Tubulin (~55 kDa) proteins detected

with anti-NS1 (1:2000; Proteintech, China) and anti-Tubulin (1:2000; Proteintech, China) antibodies. Gel-separated proteins were transferred to nitrocellulose membranes (0.2 µm pore size; Bio-Rad, Hercules, USA) by semi-dry electroblotting (1.5 mA per cm², 20 min). Membranes were incubated with a horseradish peroxidase-conjugated secondary antibody (AS09 602; 1:10000; Agrisera) and developed with Pierce™ DAB Substrate Kit (Thermo Fisher Scientific). Weight marker: Thermo Scientific™ PageRuler™ Prestained Protein Ladder, 10 to 180 kDa; catalog no. 26616. Blot images, prior to densitometry readings, were converted to grayscale with ImageJ (ImageJ v.1.49, National Institutes of Health, Maryland, USA) as follows: Image → Type → 8 bit, next: Image → Adjust → Brightness/Contrast → Auto. Cells were co-transfected with NS1, empty vector (vector), ubiquitin (Ub), and Trim21 expression plasmids, followed by treatment with DMSO (vehicle control) or cycloheximide (CHX; [concentration, e.g., 100 µg/mL]). DMSO — cells treated with DMSO; CHX — cells treated with CHX; 0, 2, 4, 6 — cells harvested at indicated hours post treatment. Tubulin was used as a loading control.

Figure S5E

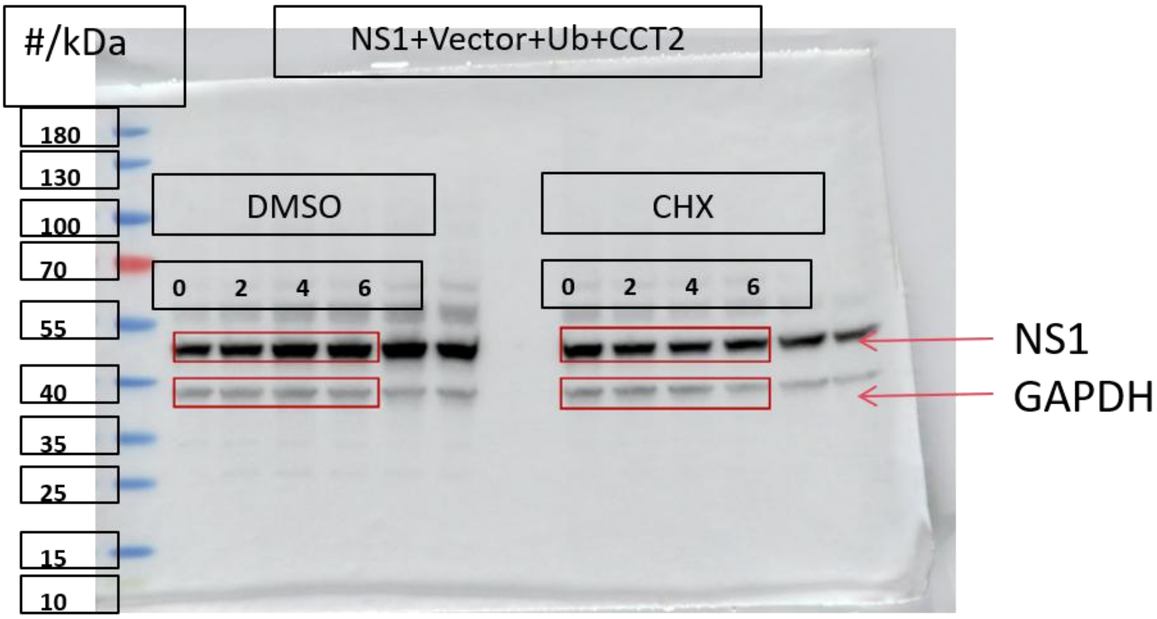

Figure S5E: Western blot membrane of NS1 (~42 kDa) and GAPDH (~36 kDa) proteins detected with anti-NS1 (1:2000; Proteintech, China) and anti-GAPDH (1:2000; Proteintech, China) antibodies. Gel-separated proteins were transferred to nitrocellulose membranes (0.2 µm pore size; Bio-Rad, Hercules, USA) by semi-dry electroblotting (1.5 mA per cm², 20 min). Membranes were incubated with a horseradish peroxidase-conjugated secondary antibody (AS09 602; 1:10000; Agrisera) and developed with Pierce™ DAB Substrate Kit (Thermo Fisher Scientific). Weight marker: Thermo Scientific™ PageRuler™ Prestained Protein Ladder, 10 to 180 kDa; catalog no. 26616. Blot images, prior to densitometry readings, were converted to grayscale with ImageJ (ImageJ v.1.49, National Institutes of Health, Maryland, USA) as follows: Image → Type → 8 bit, next: Image → Adjust → Brightness/Contrast → Auto. Cells were co-transfected with NS1, empty vector (vector), ubiquitin (Ub), and CCT2 expression plasmids, followed by treatment with DMSO (vehicle control) or cycloheximide (CHX; [100 µg/mL]). DMSO — cells treated with DMSO; CHX

— cells treated with CHX; 0, 2, 4, 6 — cells harvested at indicated hours post treatment. Tubulin was used as a loading control.

Figure S5F-1

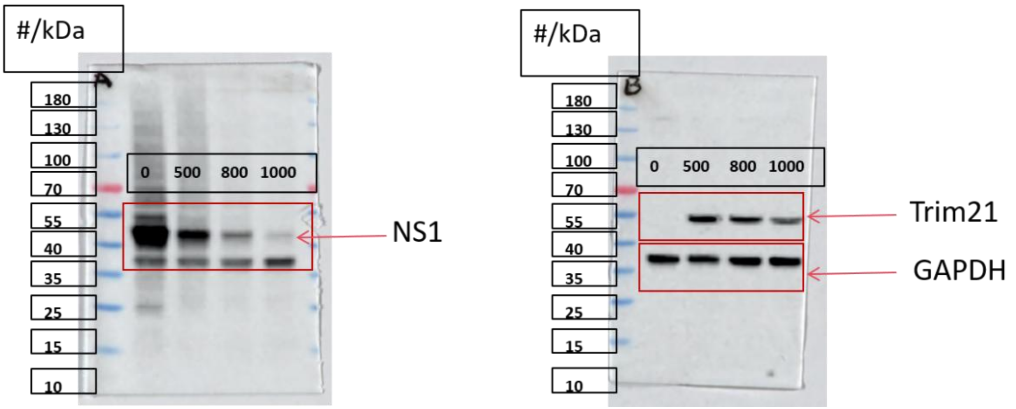

Figure S5F-1: Western blot membrane of NS1 (~42 kDa), Trim21 (~54 kDa), and GAPDH (~36 kDa) proteins detected with anti-NS1 (1:2000; Proteintech, China), anti-Trim21 (1:2000; Proteintech, China), and anti-GAPDH (1:2000; Proteintech, China) antibodies. Gel-separated proteins were transferred to nitrocellulose membranes (0.2 µm pore size; Bio-Rad, Hercules, USA) by semi-dry electroblotting (1.5 mA per cm², 20 min). Membranes were incubated with a horseradish peroxidase-conjugated secondary antibody (AS09 602; 1:10000; Agrisera) and developed with Pierce™ DAB Substrate Kit (Thermo Fisher Scientific). Weight marker: Thermo Scientific™ PageRuler™ Prestained Protein Ladder, 10 to 180 kDa; catalog no. 26616. Blot images, prior to densitometry readings, were converted to grayscale with ImageJ (ImageJ v.1.49, National Institutes of Health, Maryland, USA) as follows: Image → Type → 8 bit, next: Image → Adjust → Brightness/Contrast → Auto. Cells were transfected with increasing amounts of Trim21 plasmid (0, 500, 800, and 1000 ng), and cell lysates were harvested for Western blot analysis. GAPDH was used as a loading control.

Figure S5F-2

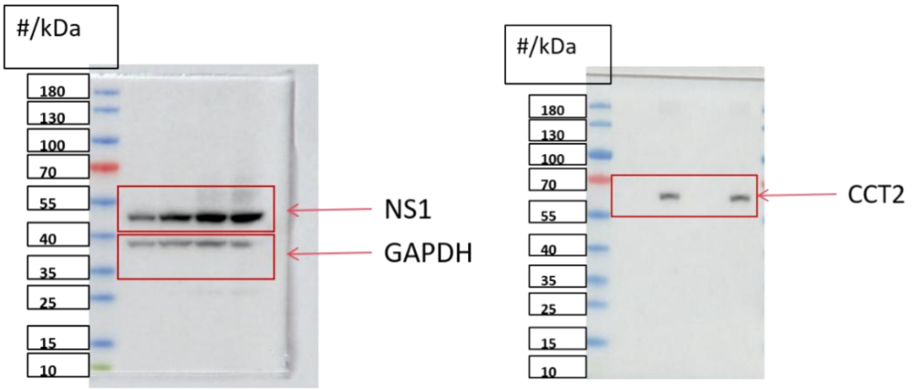

Figure S5F-2: Western blot membrane of NS1 (~42 kDa), CCT2 (~55 kDa), and GAPDH (~36 kDa) proteins detected with anti-NS1 (1:2000; Proteintech, China), anti-CCT2 (1:2000; Proteintech, China), and anti-GAPDH (1:2000; Proteintech, China) antibodies. Gel-separated proteins were transferred to nitrocellulose membranes (0.2 µm pore size;

Bio-Rad, Hercules, USA) by semi-dry electroblotting (1.5 mA per cm², 20 min). Membranes were incubated with a horseradish peroxidase-conjugated secondary antibody (AS09 602; 1:10000; Agrisera) and developed with Pierce™ DAB Substrate Kit (Thermo Fisher Scientific). Weight marker: Thermo Scientific™ PageRuler™ Prestained Protein Ladder, 10 to 180 kDa; catalog no. 26616. Blot images, prior to densitometry readings, were converted to grayscale with ImageJ (ImageJ v.1.49, National Institutes of Health, Maryland, USA) as follows: Image → Type → 8 bit, next: Image → Adjust → Brightness/Contrast → Auto. Cells were treated with DMSO (Sigma-Aldrich; vehicle control) or MG132 (MedChemExpress ,USA[10 μM]), and cell lysates were harvested for Western blot analysis. GAPDH was used as a loading control.

Figure S5G

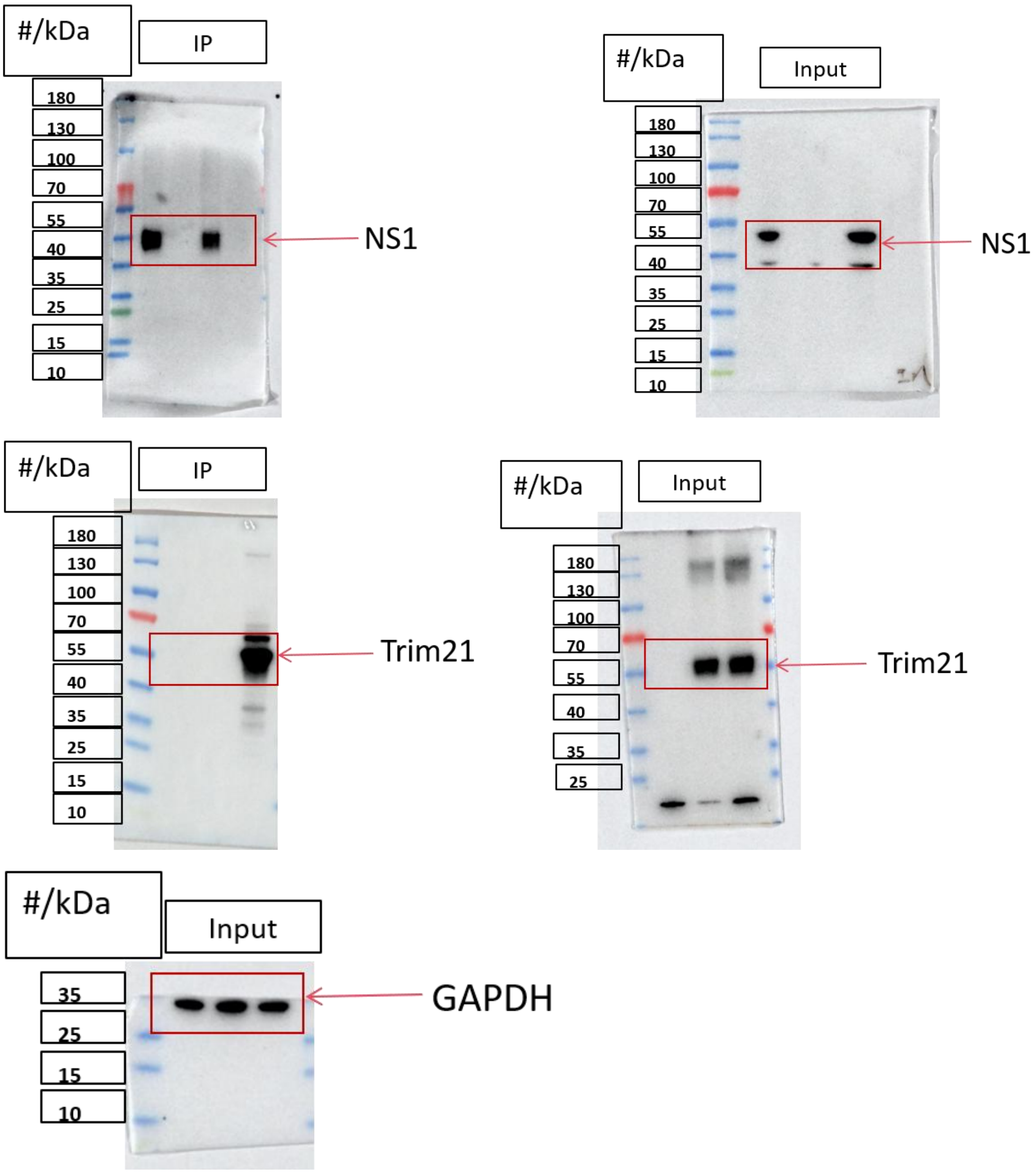

Figure S5G: Co-immunoprecipitation analysis of the interaction between NS1 and Trim21.

Cells were co-transfected with pCMV-Myc-NS1 and/or pCMV-Flag-Trim21 plasmids as indicated. Cell lysates were immunoprecipitated (IP) with anti-Myc antibody, and the immunoprecipitates were subjected to Western blotting with anti-Myc (1:2000; Proteintech, China) and anti-Flag (1:2000; Proteintech, China) antibodies to detect Myc-NS1 (~42 kDa) and Flag-Trim21 (~54 kDa), respectively. Whole-cell lysates (Input) were also analyzed by Western blotting with anti-Myc, anti-Flag, and anti-GAPDH (1:2000; Proteintech, China) 5antibodies. GAPDH (~36 kDa) was used as a loading control. Gel-separated proteins were transferred to nitrocellulose membranes (0.2 μm pore size; Bio-Rad, Hercules, USA) by semi-dry electroblotting (1.5 mA per cm², 20 min). Membranes were incubated with a horseradish peroxidase-conjugated secondary antibody (AS09 602; 1:10000; Agrisera) and developed with Pierce™ DAB Substrate Kit (Thermo Fisher Scientific). Weight marker: Thermo Scientific™ PageRuler™ Prestained Protein Ladder, 10 to 180 kDa; catalog no. 26616. Blot images were converted to grayscale with ImageJ (ImageJ v.1.49, National Institutes of Health, Maryland, USA) as follows: Image → Type → 8 bit, next: Image → Adjust → Brightness/Contrast → Auto. pCMV-Myc-NS1 — NS1 fused with Myc tag; pCMV-Flag-Trim21 — Trim21 fused with Flag tag; +, presence of transfected plasmid; –, absence of transfected plasmid.

Figure S5H

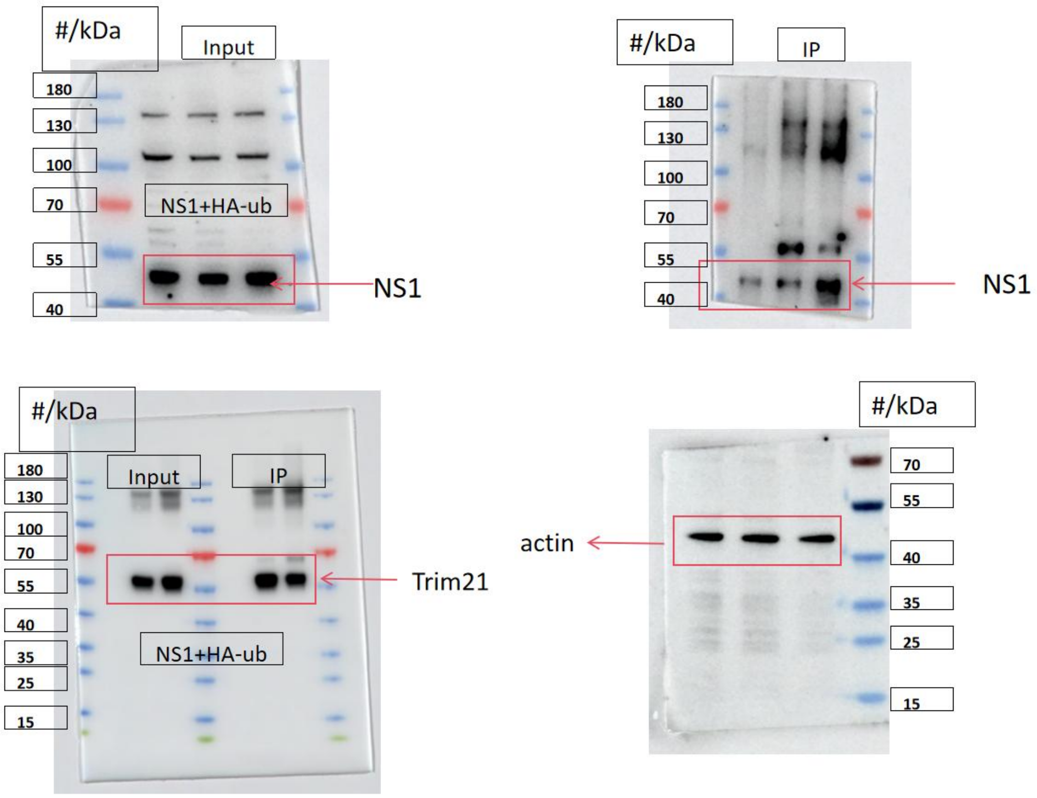

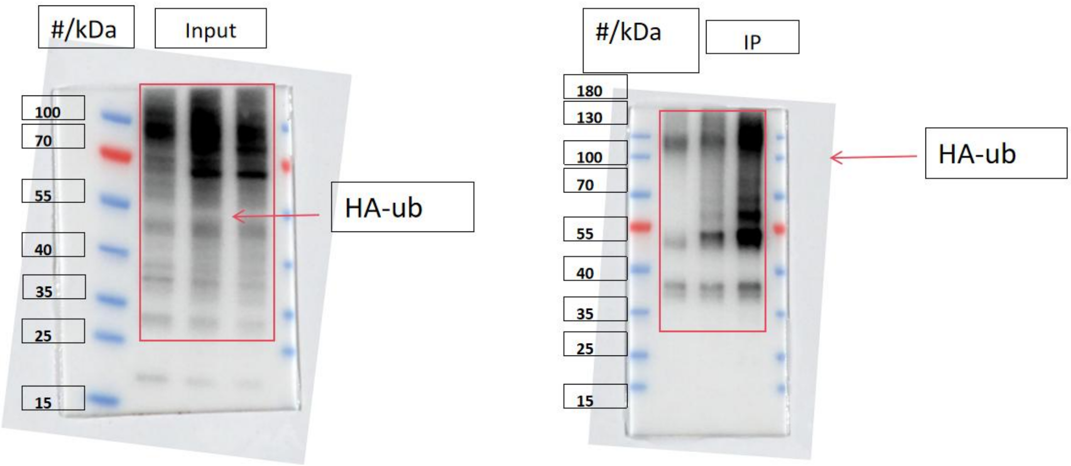

Figure S5H: Co- immunoprecipitation assay to detect TRIM21- mediated ubiquitination of NS1 in the presence or absence of MG132. Western blot membranes of Myc- NS1 (~42 kDa), Flag- TRIM21 (~54 kDa), HA- ubiquitin conjugates (high- molecular- weight ubiquitinated proteins), and β- actin (~43 kDa) proteins were detected with anti- Myc (1:2000; Nulen Biotech, China), anti- Flag (1:2000; Nulen Biotech, China), anti- HA tag (1:5000; Nulen Biotech, China), and anti- β- actin (1:2000; Proteintech, China) antibodies. Gel- separated proteins were transferred to nitrocellulose membranes (Merck Millipore, USA). Membranes were incubated with horseradish peroxidase- conjugated goat anti- mouse/anti- rabbit IgG secondary antibody (1:5000), and bands were visualized using ECL chemiluminescent substrate (Beyotime, China). #Weight marker (molecular weight in kDa): Thermo Scientific™ PageRuler™ Prestained Protein Ladder, 10 to 180 kDa; catalogue number: 26616. Blot images, prior to densitometry readings, were converted to grayscale with ImageJ (ImageJ v.1.49, National Institutes of Health, Maryland, USA) as follows: Image → Type → 8 bit, next: Image → Adjust → Brightness/Contrast → Auto. Input: whole- cell lysates before immunoprecipitation; IP: α- Myc: protein complexes immunoprecipitated using anti- Myc antibody; +, plasmid or drug treatment was applied; −, plasmid or drug treatment was omitted. High- molecular- weight ubiquitin conjugates represent ubiquitinated NS1.

Figure S6A

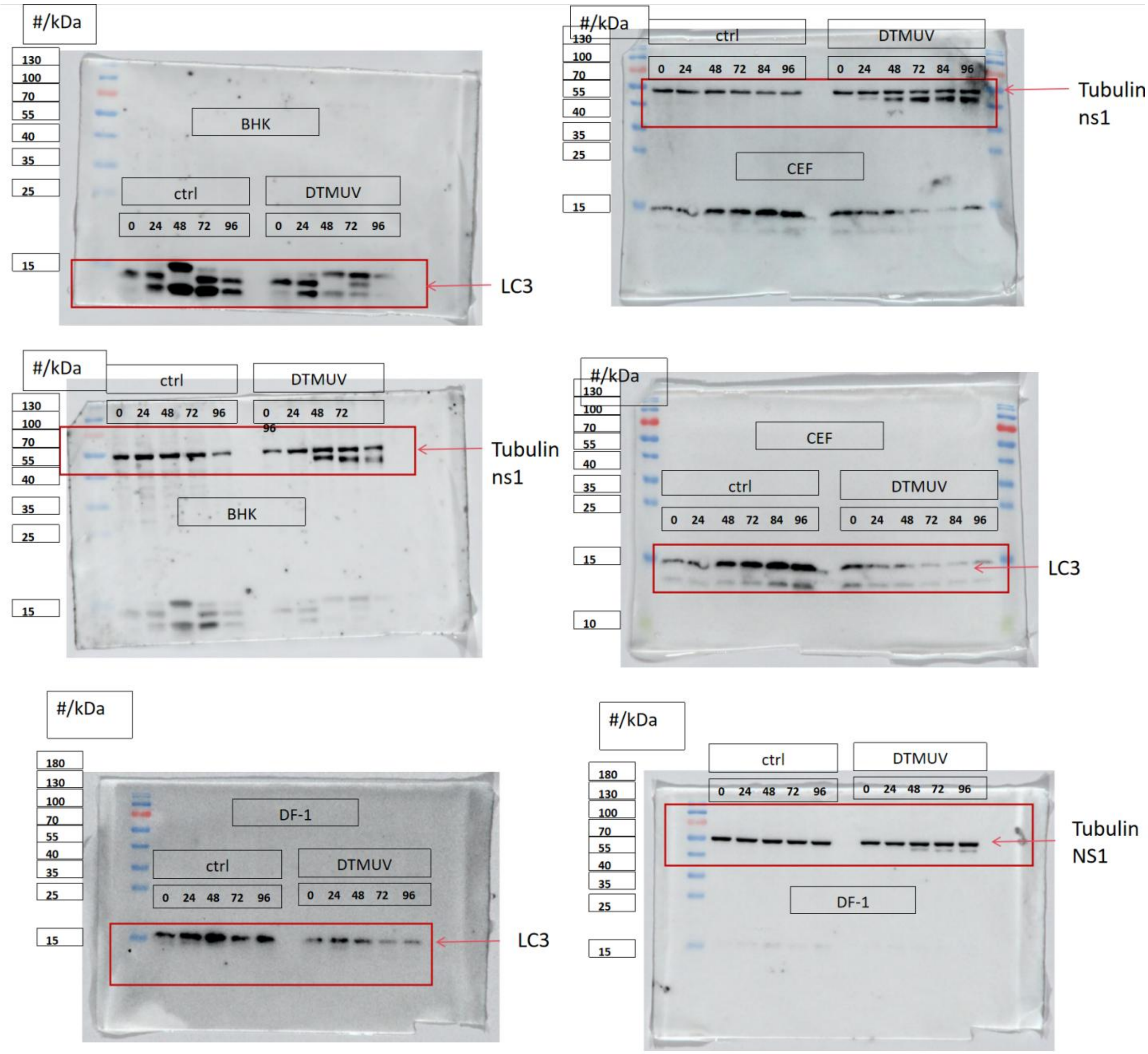

Figure S6A: Western blot analysis of NS1 and LC3 protein expression in BHK, CEF and DF- 1 cells at different time points after DTMUV infection (MOI = 1). Western blot membranes of NS1 (~42 kDa), LC3 (~16 kDa for LC3I, ~14 kDa for LC3II) and Tubulin (~55 kDa) proteins were detected with anti-NS1 monoclonal antibody (1:5000; generated in-house in our laboratory), anti-LC3 (1:2000; Proteintech, China), and anti-Tubulin (1:2000; Proteintech, China) antibodies. Gel-separated proteins were transferred to nitrocellulose membranes (Merck Millipore, USA). Membranes were incubated with horseradish peroxidase-conjugated goat anti-mouse/anti-rabbit IgG secondary antibody (1:5000), and bands were visualized using ECL chemiluminescent substrate (Beyotime, China). #Weight marker (molecular weight in kDa): Thermo Scientific™ PageRuler™ Prestained Protein Ladder, 10 to 180 kDa; catalogue number: 26616. Blot images, prior to densitometry readings, were converted to grayscale with ImageJ (ImageJ v.1.49, National Institutes of Health, Maryland, USA) as follows: Image → Type → 8 bit, next: Image → Adjust → Brightness/Contrast → Auto. MOCK: uninfected control group; DTMUV: duck Tembusu virus infected group; hpi: hours post infection. The values below the bands represent the

relative gray values of protein bands.

Figure S6C

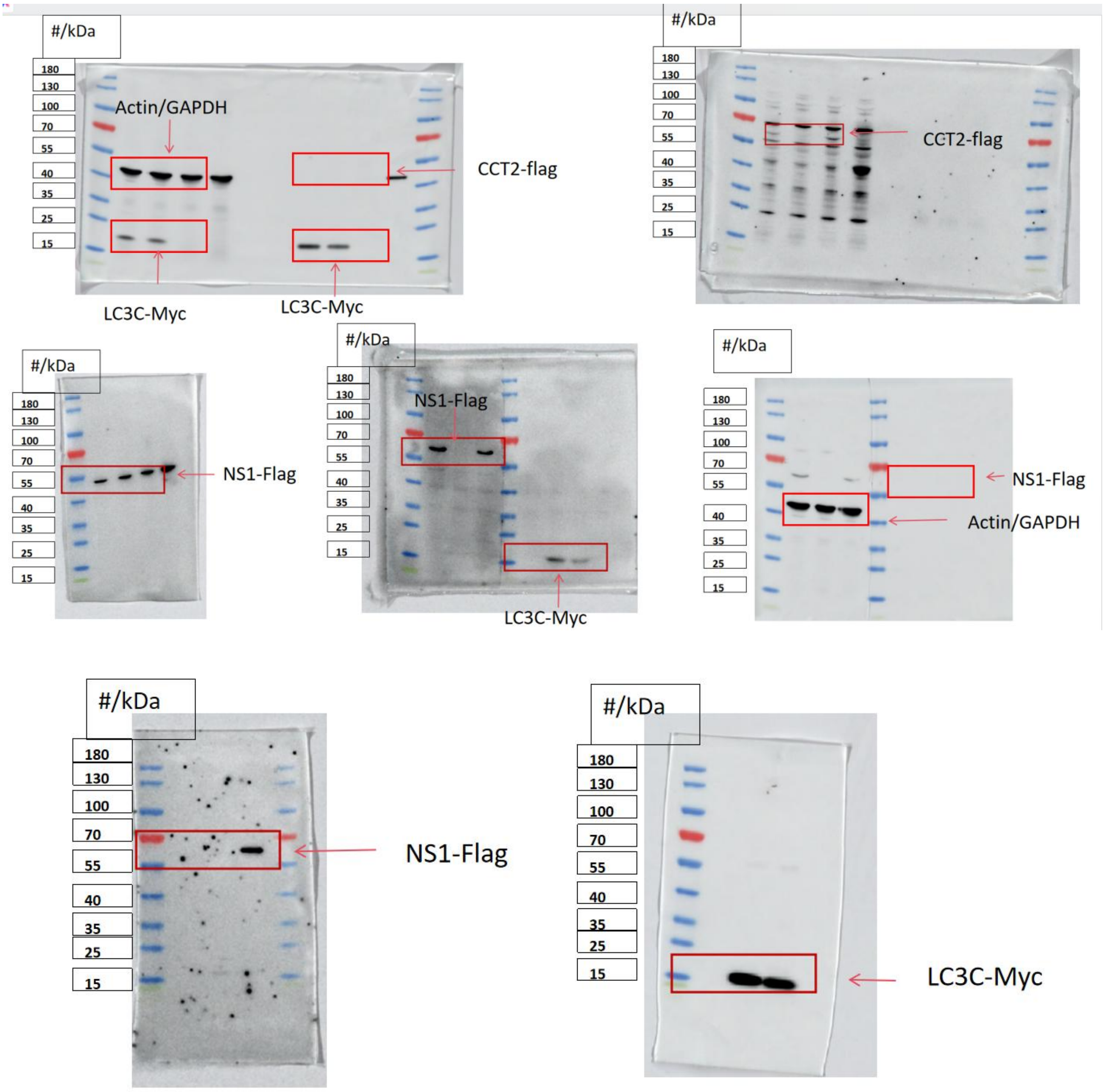

Figure S6C: Co- immunoprecipitation assay verifying the interactions among CCT2, LC3C and NS1. Western blot membranes of LC3c-Myc (~15 kDa), NS1-Flag (~42 kDa), CCT2- Flag (~60 kDa), and Actin/GAPDH (~43 kDa / ~36 kDa) proteins were detected with anti- Myc (1:2000; Nulen Biotech, China), anti- Flag (1:2000; Nulen Biotech, China), anti- Actin (1:2000; Proteintech, China) and anti- GAPDH (1:2000; Proteintech, China) antibodies. Gel-separated proteins were transferred to nitrocellulose membranes (Merck Millipore, USA). Membranes were incubated with horseradish peroxidase-conjugated goat anti-mouse/anti-rabbit IgG secondary antibody (1:5000), and bands were visualized using ECL chemiluminescent substrate (Beyotime, China). #Weight marker (molecular

weight in kDa): Thermo Scientific™ PageRuler™ Prestained Protein Ladder, 10 to 180 kDa; catalogue number: 26616. Blot images, prior to densitometry readings, were converted to grayscale with ImageJ (ImageJ v.1.49, National Institutes of Health, Maryland, USA) as follows: Image → Type → 8 bit, next: Image → Adjust → Brightness/Contrast → Auto. Input: whole-cell lysates before immunoprecipitation; IP: α-Myc: protein complexes immunoprecipitated using anti-Myc antibody; +, plasmid transfection was performed; −, plasmid transfection was omitted.

Figure S6D:

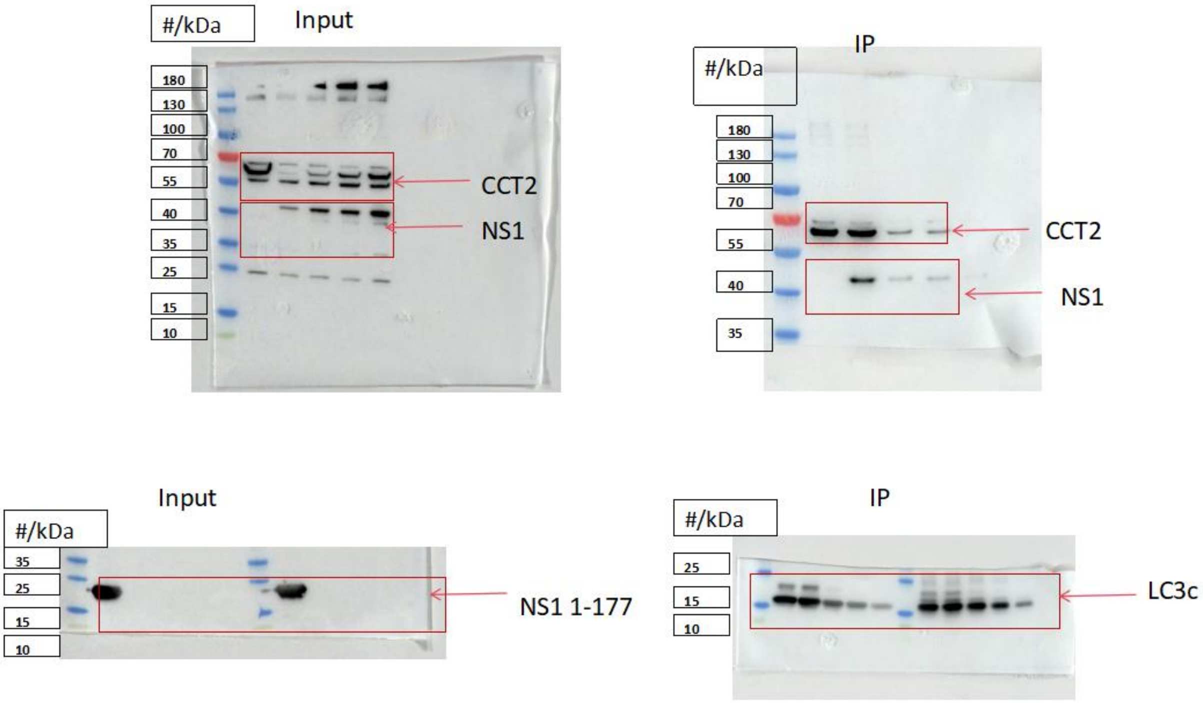

Figure S6D :Co- immunoprecipitation assay to evaluate the competitive binding of full-length NS1 to the CCT2- LC3C complex compared with $NS1_{1-177}$. Western blot membranes of Flag- CCT2 (~60 kDa), Myc- LC3C (~15 kDa), Flag- NS1 (~42 kDa), and Myc- $NS1_{1-177}$ (~22 kDa) proteins were detected with anti- Myc (1:2000; Nulen Biotech, China) and anti-Flag (1:2000; Nulen Biotech, China) antibodies. Gel- separated proteins were transferred to nitrocellulose membranes (Merck Millipore, USA). Membranes were incubated with horseradish peroxidase- conjugated goat anti- mouse/anti- rabbit IgG secondary antibody (1:5000), and bands were visualized using ECL chemiluminescent substrate (Beyotime, China). #Weight marker (molecular weight in kDa): Thermo Scientific™ PageRuler™ Prestained Protein Ladder, 10 to 180 kDa; catalogue number: 26616. Blot images, prior to densitometry readings, were converted to grayscale with ImageJ (ImageJ v.1.49, National Institutes of Health, Maryland, USA) as follows: Image → Type → 8 bit, next: Image → Adjust → Brightness/Contrast → Auto. Input: whole- cell lysates before immunoprecipitation; IP: α- Myc: protein complexes immunoprecipitated using anti- Myc antibody. The gradient triangle indicates increasing transfection amounts of Flag- NS1 plasmid (0.5, 1, 2, 3 μg).
